# HuR Confers Resistance of Hepatocellular Carcinoma to Lenvatinib by Inhibiting Ferroptosis

**DOI:** 10.1111/jcmm.71423

**Published:** 2026-11-03

**Authors:** Siren Feng, Fang Wang, Huayi Wu, Xinran Lin, Xue Qi, Kun Liu, Qiushi Yin, Bo Lin, Mengsen Li, Mingyue Zhu

**Affiliations:** ^1^ Key Laboratory of Tropical Translational Medicine, Ministry of Education, Hainan Provincial Key Laboratory of Carcinogenesis and Intervention, School of Basic Medical Sciences Hainan Medical University Haikou Hainan Province P.R. China; ^2^ Department of Medical Oncology The Second Affiliated Hospital of Hainan Medical University Haikou Hainan Province P.R. China

**Keywords:** drug resistance, ferroptosis, hepatocellular carcinoma, HuR, lenvatinib

## Abstract

This study aim to explore whether human antigen R (HuR) inhibits ferroptosis confers resistance to lenvatinib in hepatocellular carcinoma (HCC). Bioinformatics analysis and immunofluorescence detection of 30 HCC tissue samples were used to verify the clinical correlation between HuR and glutathione peroxidase 4 (GPX4), establish a lenvatinib‐resistant HCC cell line, and evaluate the cell phenotypes and ferroptosis indicators (Fe^2+^, reactive oxygen species (ROS), lipid peroxidation, glutathione (GSH)) through functional experiments. The HuR‐GPX4 axis was analysed by genetic intervention methods and verified through xenograft mouse tumour models. Co‐expression of HuR/GPX4 in HCC tissues was associated with poor prognosis. Under lenvatinib treatment, the drug‐resistant cell lines showed increased malignant behaviour and ferroptosis resistance characteristics (decreased ferrous ions, ROS, lipid peroxidation, and increased GSH) in HCC cells, as well as upregulation of lenvatinib targeting vascular endothelial growth factor receptor 1 (VEGFR1)/fibroblast growth factor receptor 3 (FGFR3). HuR promotes the expression of GPX4 via cytoplasmic translocation. Interference with the expression of HuR could reverse the drug resistance phenotype and increase ferroptosis in lenvatinib‐induced HCC; overexpression of GPX4 partially restored drug resistance. HuR is an important molecule for conferring resistance to lenvatinib therapy and may be involved in inhibiting ferroptosis by upregulating the expression and activity of GPX4. This study provides a new strategy for targeting patients with HCC.

**Abbreviations**
ACSL4: acyl‐CoA synthetase long‐chain family member 4
EGFR: epidermal growth factor receptor
ERK: extracellular‐signal regulated kinase
FGFR: fibroblast growth factor receptor
FTH1: ferritin heavy chain 1
GPX4: glutathione peroxidase 4
GSH: glutathione
GSSG: glutathione disulfide
HCC: hepatocellular carcinoma
HuR: human antigen R
ICIs: immune checkpoint inhibitors
LDHA: lactate dehydrogenase A
MAPK: mitogen‐activated protein kinase
MDA: malonaldehyde
MDR: multidrug‐resistant
MEK: mitogen‐activated protein kinase kinase
MRP1: multidrug resistance‐associated protein 1
PUFAs: polyunsaturated fatty acids
ROS: reactive oxygen species
RTK: receptor tyrosine kinase
SLC7A11: cystine/glutamate antiporter solute carrier family 7 member 11
TFR1: transferrin receptor 1
TKIs: tyrosine kinase inhibitors
VEGF: vascular endothelial growth factor
VEGFR: vascular endothelial growth factor receptor

## 1 Introduction

Hepatocellular carcinoma (HCC) is a common primary malignant tumour in the liver, the sixth most common malignant tumour, and the fourth leading cause of cancer‐related deaths worldwide [1]. HCC accounts for approximately 80%–90% of all liver malignancies [2]. Although early stage HCC can usually be treated through surgical resection or local therapy, including transplantation and chemoembolization [3], because of the lack of obvious early symptoms in patients with liver cancer and the absence of early diagnostic markers and therapeutic targets, many patients diagnosed with liver cancer have reached an advanced stage of the disease (HCC with liver metastasis or local HCC with portal vein invasion) [4], missing the optimal surgical time and thus are not suitable for local treatment [5]. At present, treatment regimens for patients with advanced liver cancer usually adopt systemic therapies, such as tyrosine kinase inhibitors (TKIs) and immune checkpoint inhibitors (ICIs) [2]. Lenvatinib and sorafenib are common TKIs that inhibit HCC progression by suppressing various receptor tyrosine kinases (RTK) [6]. Compared with sorafenib, lenvatinib has a higher objective response rate and disease control rate and has better safety and tolerability in HCC patients [7].

Lenvatinib is an oral multi‐receptor tyrosine protein kinase inhibitor. It mainly inhibits vascular endothelial growth factor receptor 1–3 (VEGFR1‐VEGFR3), fibroblast growth factor receptor 1–4 (FGFR1‐FGFR4), platelet‐derived growth factor receptor α (PDGFRα), KIT receptor tyrosine kinase (KIT), and transfection rearrangement protein (RET). Therefore, inhibition of the mitogen‐activated protein kinase (MAPK) cascade further suppresses tumour angiogenesis and the development of oncogenic signalling pathways [8]. Studies have shown that lenvatinib resistance is related to the enrichment of cancer stem cells, remodelling of the tumour immune microenvironment, epithelial‐mesenchymal transition, and activation of multiple downstream signalling pathways such as RAS/MEK/ERK and epidermal growth factor receptor (EGFR) [9]. This suggests that the resistance mechanism of lenvatinib is diverse, and there are still effective downstream intervention targets that target the resistance pathway. Therefore, it is necessary to explore the potential resistance mechanism of lenvatinib, which plays a very important role in therapeutic targets and improvement of the prognosis of patients with HCC.

Studies have found that lenvatinib can reduce the activity of glutathione peroxidase 4 (GPX4) protein by inhibiting FGFR4, promoting the accumulation of lipid peroxides, thereby leading to ferroptosis in hepatoma cells [10]. This indicates that the stability of GPX4 plays an important role in the efficacy of lenvatinib‐resistant HCC cells. In addition, lenvatinib significantly reduced the expression of GPX4 in the mouse aorta and human umbilical vein endothelial cells (HUVEC) and increased lipid peroxidation, lactate dehydrogenase (LDH) release, and malondialdehyde (MDA) levels in HUVEC, leading to ferroptosis of endothelial cells [11]. GPX4 is an evolutionarily highly conserved enzyme that can limit the intracellular spread of lipid peroxidation by reducing perphospholipid and cholesterol hydroperoxides to non‐toxic lipid alcohols [12]. This process can prevent cell death caused by the formation of toxic lipid reactive oxygen species (ROS) in a Fe^2+^‐dependent manner, namely ferroptosis [13].

The RNA‐binding protein human antigen R (HuR) primarily stabilizes protein expression by translocating from the nucleus to the cytoplasm to bind the 3′‐UTR of mRNA and prolong the half‐life of the bound mRNA transcripts [14]. HuR controls the proliferation of HCC cells through various mechanisms. For instance, HuR stabilizes the transcripts of the cell cycle regulators CCNA1, CCNB1, and CDK3 by translocation to the cytoplasm of HCC cells, promoting the proliferation of cancer cells [15, 16, 17]. The migration and invasion abilities of HCC cells are important determinants for evaluating the survival of patients. Hepatitis B virus‐encoded X protein (HBX) increases the expression of human epidermal growth factor receptor‐2 (HER2) by inducing the expression of HuR, thereby promoting the migration ability of HCC cells [18].

This study aimed to explore the mechanism by which HuR upregulates the expression of GPX4 by stabilizing GPX4 mRNA, thereby inhibiting ferroptosis in HCC cells and promoting lenvatinib resistance. First, we detected the expression differences of HuR and GPX4 in HCC tissues and adjacent tissues through bioinformatics analysis and evaluated the correlation between the two and their association with the prognosis of HCC patients. Subsequently, through in vitro cell experiments and in vivo animal models, the role of HuR in the ferroptosis regulatory pathway mediated by GPX4 in liver cancer drug resistance was systematically studied to clarify the effect of the HuR‐GPX4 signalling axis on the sensitivity of HCC cells to lenvatinib.

## 2 Methods

### 2.1 Clinical Samples

The RNA sequence (RNA‐SEQ) data of 424 HCC cases were downloaded from The Cancer Genome Atlas (TCGA) database, including 374 HCC samples and 50 samples of adjacent normal liver tissues. In addition, tissue samples from 30 HCC patients were collected from the Second Affiliated Hospital of Hainan Medical University (January 2023 to December 2024), and all experiments were approved by the Experimental Animal Ethics and Use Committee of Hainan Medical University (Ethics Committee Approval Certificate (No. HYLL‐2023‐150)). The inclusion criteria for the samples were as follows: (1) age between 18 and 80 years, (2) at least 6 months after the diagnosis of HCC, (3) willingness to accept follow‐up visits, and (4) no anti‐cancer drug treatment or traditional Chinese medicine treatment in the past 6 months. This study involved the detection of protein expression in human liver cancer samples and animal experiments, strictly in accordance with the Declaration of Helsinki.

### 2.2 Cell Line

The cell lines selected for this experiment were human hepatoma cell lines Huh7, Bel7404, PLC/PRF/5, HLE, and the human normal hepatocyte cell line THLE‐2. All the cells were purchased from the Shanghai Institute of Cell Biology, Chinese Academy of Sciences (Shanghai, China).

### 2.3 Experimental Reagents

Dulbecco's Modified Eagle Medium (DMEM), fetal bovine serum (Gibco, USA); Lenvatinib (MCE, USA); CCK8 detection reagent (Tongren Chemical Research Institute, Japan); Cell ferrous colorimetric test kit; reactive oxygen species fluorescence test kit (red) (Wuhan Yilerite Biotechnology Co. Ltd); Lipid oxidation detection kit, GSH and GSSG detection kit (Biyuntian Biotechnology Co. Ltd); GPX4, ACSL4, TFR1, Ferritin heavy chain, SLC7A11/xCT, Alexa Flour 594, Alexa Flour 488 (Wuhan Sanying Biotechnology Co. Ltd); VEGFR1, FGFR3 (Hangzhou Hua'an Biotechnology Co. Ltd); HuR (Santa Cruz, USA); 812 Embedding Agent (SPI); Osmium acid (Ted Pella Inc); Annexin V‐APC/PI Double Staining Apoptosis Detection Kit (Jiangsu Kaiji Biotechnology Co. Ltd); RNA extraction kit, reverse transcription kit, Fluorescence quantitative PCR kit (Beijing Baori Biotechnology Co. Ltd).

### 2.4 Bioinformatics Analysis

Using the TIMER HuR and GPX4 database (http://timer.cistrome.org/) analysis for a variety of differences in tumour expression. The rank sum test and *t*‐test were used to analyse differences in gene expression between the tumour and normal groups. The correlation between the two genes was analysed using Spearman's correlation.

### 2.5 Collection of Experimental Tissue Specimens

Tumour tissues and adjacent normal liver tissues of 30 HCC patients who were hospitalized between January 2023 and December 2024 were collected. The adjacent normal liver tissues were located more than 2 cm away from the boundary between the lesion and normal liver tissues. The collected specimen tissues were immersed in 4% paraformaldehyde fixation solution and made into tissue microarrays.

### 2.6 Organize Chip Technology

Clinical samples were fixed with 4% paraformaldehyde to prepare the tissue microarrays. Fixation → dehydration → paraffin embedding → sectioning → antigen repair → primary antibody (4°C overnight) → fluorescent secondary antibody → DAPI mounting → imaging.

### 2.7 Construction of Lenvatinib‐Resistant Cell Lines

Huh7 and Bel7404 cells were induced with a gradient increase in lenvatinib concentration for 6 months, and drug‐resistant strains (Huh7‐R and Bel7404‐R) were obtained.

### 2.8 Cell Scratch Experiment

Inoculate 5 × 10^5^ cells into each well and spread the plates evenly. Scratch with a sterile pipette tip and capture a baseline image (T0). The drug‐containing medium was replaced and the cells were placed in a CO_2_ incubator for culturing. The scratch areas were photographed at 24, 48, and 72 h to quantitatively analyse the cell migration ability.

### 2.9 Cell Plate Cloning Experiment

Cells were inoculated at a rate of 700–1500 per well and routinely cultured in a medium containing lenvatinib for 14–16 days until clones were visible to the naked eye. The cells were fixed with 4% paraformaldehyde for 30 min, stained with 0.1% crystal violet for 30 min, rinsed with running water, and dried. After microscopic imaging, the number of clones was counted and the clone formation rate was calculated.

### 2.10 Cell Counting Kit‐8 (CCK8) Analysis

The cells were inoculated into 96‐well plates (100 μL/well) according to the blank, control, and gradient drug concentration groups. After drug treatment for 24, 48, and 72 h, fresh culture medium was replaced, and CCK8 (10 μL/well) was added. The cells were incubated in the dark for 30–60 min, and the absorbance at 450 nm was measured using an enzyme‐linked immunosorbent assay (ELISA) reader.

### 2.11 Cellular Apoptosis Detection

Drug‐containing medium was added to the cells, which were digested into a single‐cell suspension using trypsin without ETDA. Approximately 1–5 × 10^5^ cells were collected from each well. Add 500 μL of binding buffer and gently resuspend the cells to obtain a single‐cell suspension. Annexin V (5 μL) and PI dye (5 μL) were added and mixed evenly. Reaction at room temperature in the dark for 10–15 min. The analysis and detection were performed on a flow cytometer within 1 h. The apoptotic rate was calculated as the sum of early apoptotic (Annexin V‐APC^+^/PI^−^) and late apoptotic (Annexin V‐APC^+^/PI^+^) cells divided by the total number of acquired cells (≥ 10,000 events per sample), expressed as a percentage.

### 2.12 Western Blotting

The cells were collected and lysed with lysis buffer containing protease/phosphatase inhibitors. Centrifuge (12,000 rpm, 15 min) and the supernatant was aliquoted. The protein concentration was determined using the BCA method. After diluting the sample in equal amounts, it was mixed with 4× SDS Loading Buffer (3:1) and denatured at 100°C for 10 min. SDS‐PAGE electrophoresis (pre‐prepared gel, 20–80 μg/well), transferred to PVDF membrane (transfer at constant pressure at 4°C for 1.5–2 h). Incubate at 37°C for 15 min; the primary antibody was incubated at 4°C overnight, the secondary antibody was incubated at 37°C for 1 h, and ECL was used for imaging.

### 2.13 Intracellular Ferrous Ion Detection

Prepare the standard curve, collect the cells to be tested, add 0.2 mL of reagent one for every 1 × 10^6^ cells, lyse in an ice box for 10 min, centrifuge at 15,000 *g* for 10 min, and retain the supernatant. Standard, control, and sample wells were tested. Standard substances of different concentrations (80 μL) were added to the standard wells; 80 μL of reagent two (control solution) was added to the control well; 80 μL of the sample was added to the sample wells to be tested, and finally, 80 μL of reagent three (chromogenic solution) was added.

### 2.14 Intracellular ROS Detection

2 × 10^5^ cells into six‐well plates. After adhesion, add 10 μmol/L fluorescent probe working solution and incubated in the dark for 30 min (37°C). Trypsin digestion was performed, centrifuged at 300× *g* for 5 min, and the cells were collected. After resuspension in PBS and filtration through a 40 μm filter screen, the fluorescence signal was detected by flow cytometry (PE channel).

### 2.15 Detection of Malonaldehyde (MDA) in HCC Cells

Cell groups were established, cells were collected, whole proteins were extracted, and protein concentration was determined. The corresponding MDA detection working solution was prepared according to the number of samples (the sample dosage required for one detection was 150 μL TBA dilution, 50 μL TBA stock solution, and 3 μL antioxidant). Reaction system: 200 μL of MDA detection working solution was added to 100 μL of the sample to be tested. After the system was prepared, it was heated and reacted at 100°C for 20 min. After the reaction, the mixture was cooled and centrifuged at 1000× *g* at room temperature for 10 min. Then, 200 μL of the supernatant was added to a 96‐well plate and the absorbance was measured at 532 nm.

### 2.16 Intracellular Glutathione (GSH) Detection

Collect The cells, lysed them at a ratio of 1:3 (cell precipitate: protein removal reagent M), rapidly frozen, thawed twice at 37°C in liquid nitrogen, and centrifuged at 4°C to obtain the supernatant. GSSG sample preparation: For every 100 μL of sample, 20 μL of GSH clearance auxiliary solution and 4 μL of GSH clearance working solution were added in sequence, mixed well, and set aside for later use. The total GSH and GSSG samples were added to 96‐well plates. The total GSH detection solution (150 μL) and 0.5 mg/mL NADPH (50 μL) were added to each well. The mixture was mixed thoroughly and allowed to react at room temperature for 25 min. Absorbance was measured at 412 nm using an enzyme‐linked immunosorbent assay reader.

### 2.17 Cellular Immunofluorescence Observation

The cells were inoculated into confocal dishes and treated with lenvatinib at the same concentration for 48 h on the following day. PBS washing → Fixation with 4% paraformaldehyde for 30 min (500 μL) → PBS washing → sealing with blocking solution for 15 min → primary antibody at 4°C overnight → secondary antibody incubated at 37°C in the dark for 1 h → PBS washing → DAPI sealing, confocal microscopy imaging.

### 2.18 Nuclear and Mass Separation and Western Blotting Assay

Extraction of nucleoplasmic proteins by ice lysis: The cell precipitate was pre‐cooled and lysed with CER I, incubated in an ice bath, mixed evenly with CER II vortex, centrifuged at 4°C (16,000 *g*, 5 min), and the supernatant (cytoplasmic protein) was aliquoted and stored at −80°C. The pre‐cooled NER lysis buffer was added to the precipitate and vortexed (15 s each time, with an interval of 10 min, for a total of 40 min) → centrifuge (16,000 *g*, 10 min), and the supernatant (nucleoprotein) was collected. The distribution of nucleoplasmic proteins was detected by Western blotting.

### 2.19 Construction of the BALB/c Nude Mouse Tumour Model

Cells in the logarithmic growth phase were digested with trypsin, centrifuged (1800 rpm for 10 min), and resuspended in PBS. The transplanted tumour model of BALB/c female nude mice (3 weeks old) was constructed by subcutaneous injection (5 × 10^6^ cells/200 μL). After the tumour had grown to an appropriate volume, 10 mg/kg lenvatinib was administered by gavage. After the treatment was completed, the mice were sacrificed, and the weight and volume of the tumours were measured. Tumour tissues were subjected to immunohistochemical and western blot experiments. We hereby declare that the animal experiments involved in this research were approved by the Research Ethics Committee of Hainan Medical University and strictly adhered to the requirements of international animal welfare and the Helsinki Declaration of International Scientific Research.

### 2.20 Immunohistochemistry Analysis

After the tumour tissues were fixed with 4% paraformaldehyde, they were dehydrated with gradient ethanol and embedded in paraffin. The sections were subjected to xylene dewaxing, gradient ethanol rehydration, microwave antigen repair with citric acid buffer (pH 6.0), 3% H_2_O_2_ inactivation of endogenous enzymes, and 5% BSA blocking. Primary antibody 4°C overnight → secondary antibody 1 h at room temperature → DAB colour development, haematoxylin re‐staining the nucleus → ethanol gradient dehydration → xylene transparency → neutral gum sealing, microscope imaging.

### 2.21 Transmission Electron Microscope Observation

The cell precipitate was collected and fixed with 2.5% glutaraldehyde at 4°C for 4 h. Cells were pre‐embedded with 1% agarose and fixed with 1% osmium acid at room temperature in the dark for 2 h. Gradient ethanol dehydration → acetone displacement → 812 epoxy resin gradient permeation → 2% uranium acetate dark staining for 8 min → 2.6% lead citrate (CO_2_‐free) staining for 8 min → observation by transmission electron microscopy.

### 2.22 qRT‐PCR

Extract RNA: Add an appropriate amount of lysis Buffer R and pipette until it is clear. DNA removal: Centrifuge for 1 min × 2 times, discard the column, and retain the filtrate. The filtrate was mixed with an equal volume of 70% ethanol, transferred to an RNA collection column, and centrifuged for 1 min × 2 times. Cleaning: Add 500 μL of Buffer RWA (12,000 rpm × 30 s) and 600 μL of Buffer RWB (12,000 rpm × 30 s × 2 times) sequentially. Elution: Add 70 μL of enzyme‐free water for elution, centrifuged to collect RNA, measure the concentration, and store at −80°C. Removal of genomic DNA: 2.0 μL 5× gDNA Eraser Buffer; 1.0 μL gDNA Eraser 1 μg Total RNA Fill up with enzyme‐free water to 10 μL. Reverse transcription: Reaction system (10 μL of the reaction solution from the previous step; 1 μL RT Enzyme Mix 1 μL RT Primer Mix 4 μL 5× PrimeScript Buffer 2 4 μL of enzyme‐free water; program: 37°C for 15 min → 85°C for 5 s → 4°C). qPCR: Reaction system (10 μL SYBR qPCR Master Mix; 0.4 μL forward/reverse primers; 2 μL cDNA template 7.2 μL of enzyme‐free water, procedure: Pre‐denaturation at 95°C for 30 s; amplification for 40 cycles (95°C for 5 s → 60°C for 30 s); dissolution curve analysis (95°C → 60°C → 95°C)).

### 2.23 Statistical Analysis

Data normality was assessed by Shapiro–Wilk test. Normally distributed data are shown as mean ± SD; comparisons between two groups used two‐tailed unpaired Student's *t*‐test, and multiple group comparisons used one‐way or two‐way ANOVA followed by Tukey's post hoc test. Non‐normally distributed data were analysed by Mann–Whitney *U* test or Kruskal–Wallis test with Dunn's correction. Multiple comparisons across different ferroptosis parameters were corrected using the Benjamini–Hochberg false discovery rate method. GraphPad Prism 9.0 was used. *p* < 0.05 was considered significant.

## 3 Results

### 3.1 The Expressions of HuR and GPX4 Increase Positive Correlated With HCC

The analysis results of the timer database online showed that compared with the normal tissues corresponding to various cancers, the expression of GPX4 was upregulated in various cancer tissues, and the expression of GPX4 in liver cancer was significantly higher than that in adjacent tissues (*p* < 0.001); moreover, compared with adjacent tissues, the expression level of HuR was significantly upregulated in various cancer tissues, and the expression level of HuR in liver cancer was also significantly higher than that in adjacent tissues (*p* < 0.001) (Figure 1A,B). The expression levels of HuR and GPX4 in liver cancer tissues and adjacent normal liver tissues showed that the expression levels of HuR and GPX4 in HCC tissues were higher than those in adjacent tissues (*p* < 0.001; Figure 1C,D). Spearman correlation analysis showed that HuR was positively correlated with GPX4 (*r* = 0.153, *p* = 0.003) (Figure 1E). Multiplex fluorescence staining of tissue microarrays showed that the densities of positive cells of HuR and GPX4 in HCC tissues were higher than those in adjacent tissues (Figure 1F,G). High HuR expression was observed in 27 cases (90%), whereas only 3 cases (10%) of the corresponding adjacent normal tissues exhibited high expression. The results indicated that the expression of HuR and GPX4 in HCC tumour tissues was higher than that in the adjacent tissues.

**FIGURE 1:**
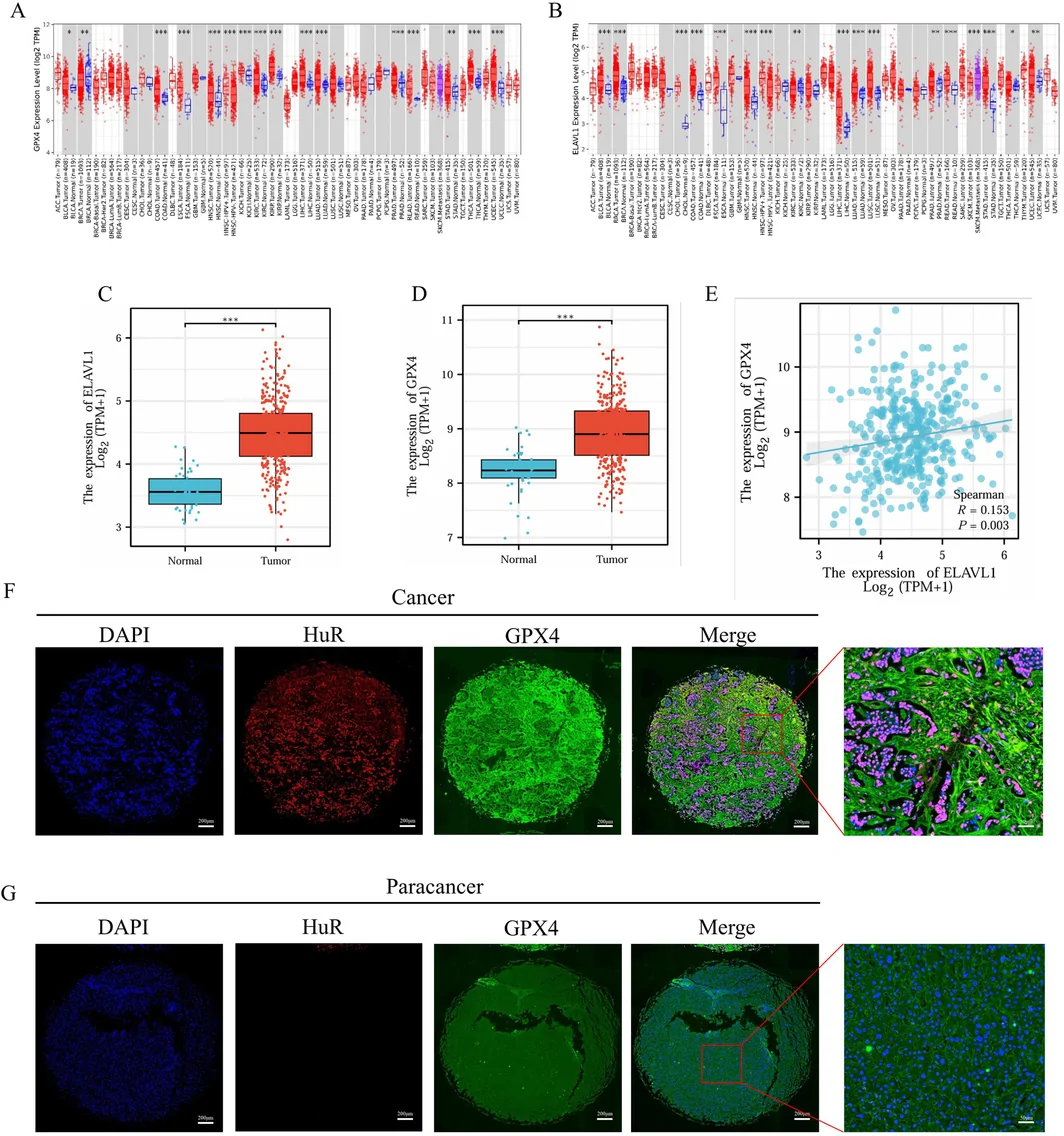
The expression of HuR and GPX4 in HCC. (A) The expression differences of GPX4 in various cancers and their corresponding adjacent normal groups. (B) Differences in the expression of HuR in various cancers and their corresponding adjacent normal groups. (C) The box plot shows the expression differences of HuR in HCC and corresponding adjacent tissues in the TCGA database. (D) The box plot shows the expression differences of GPX4 in HCC and corresponding adjacent normal tissues in the TCGA database. (E) Scatter plot of correlation between HuR and GPX4. (F) The expression of HuR and GPX4 in cancer tissues. (G) The expression of HuR and GPX4 in corresponding adjacent normal tissues; the lengths of the scales are 200 and 50 μm. DAPI, 4′,6‐diamidino‐2‐phenylindole; GPX4, glutathione peroxidase 4; HuR, human antigen R. Data are presented as mean ± SD from three independent experiments. Statistical comparisons between two groups were performed using unpaired two‐tailed Student's *t*‐test. For multiple groups, one‐way ANOVA with Tukey's post hoc test was used. *p < 0.05; **p < 0.01; ***p < 0.001; ^ns^ *p* > 0.05.

### 3.2 Construction of Lenvatinib‐Resistant Cell Lines and the Association Between Lenvatinib Resistance in Liver Cancer

Successfully constructed lenvatinib‐resistant cell lines (Bel7404‐R and Huh7‐R). Bel7404‐R elongated antennae and increased intercellular segregation (Figure 2A); Huh7‐R cells with regular morphology, clustered into clusters, and increased local cell density (Figure 2B). The drug resistance of Bel7404‐R (IC_50_ = 35.32 μM) was 1.7 times higher than that of Bel7404 (IC_50_ = 20.57 μM) (*p* < 0.01); The drug resistance of Huh7‐R (IC_50_ = 24.63 μM) was 2.8 times higher than that of Huh7 (IC_50_ = 4.378 μM) (*p* < 0.001) (Figure 2C,D). The scratch healing rates of Bel7404‐R and Huh7‐R cells were significantly higher than those of parental cells (*p* < 0.01) (Figure 2E,F). The number of cloned colonies of drug‐resistant strains increased significantly (*p* < 0.01) (Figure 2I,J). The total apoptosis rate of Bel7404‐R (10.54%) was significantly lower than that of Bel7404 (46.14%; *p* < 0.01). The total apoptosis rate of Huh7‐R cells (37.36%) was significantly lower than that of Huh7 cells (51.66%, *p* < 0.05) (Figure 2G,H). The expression of drug resistance‐related proteins VEGFR1 and FGFR3 in Bel7404‐R and Huh7‐R cells was significantly upregulated compared to that in the parental cells (Figure 2K,L). These results indicated that the lenvatinib‐resistant strains Bel7404‐R and Huh7‐R were successfully constructed. The drug‐resistant cells showed enhanced migration, clone formation ability, and anti‐apoptotic properties, accompanied by a high expression of VEGFR1/FGFR3. Since VEGFR1/FGFR3 is the target of lenvatinib, this suggests that the high expression of VEGFR1/FGFR3 in drug‐resistant cell lines is beneficial for resisting the effect of lenvatinib.

**FIGURE 2:**
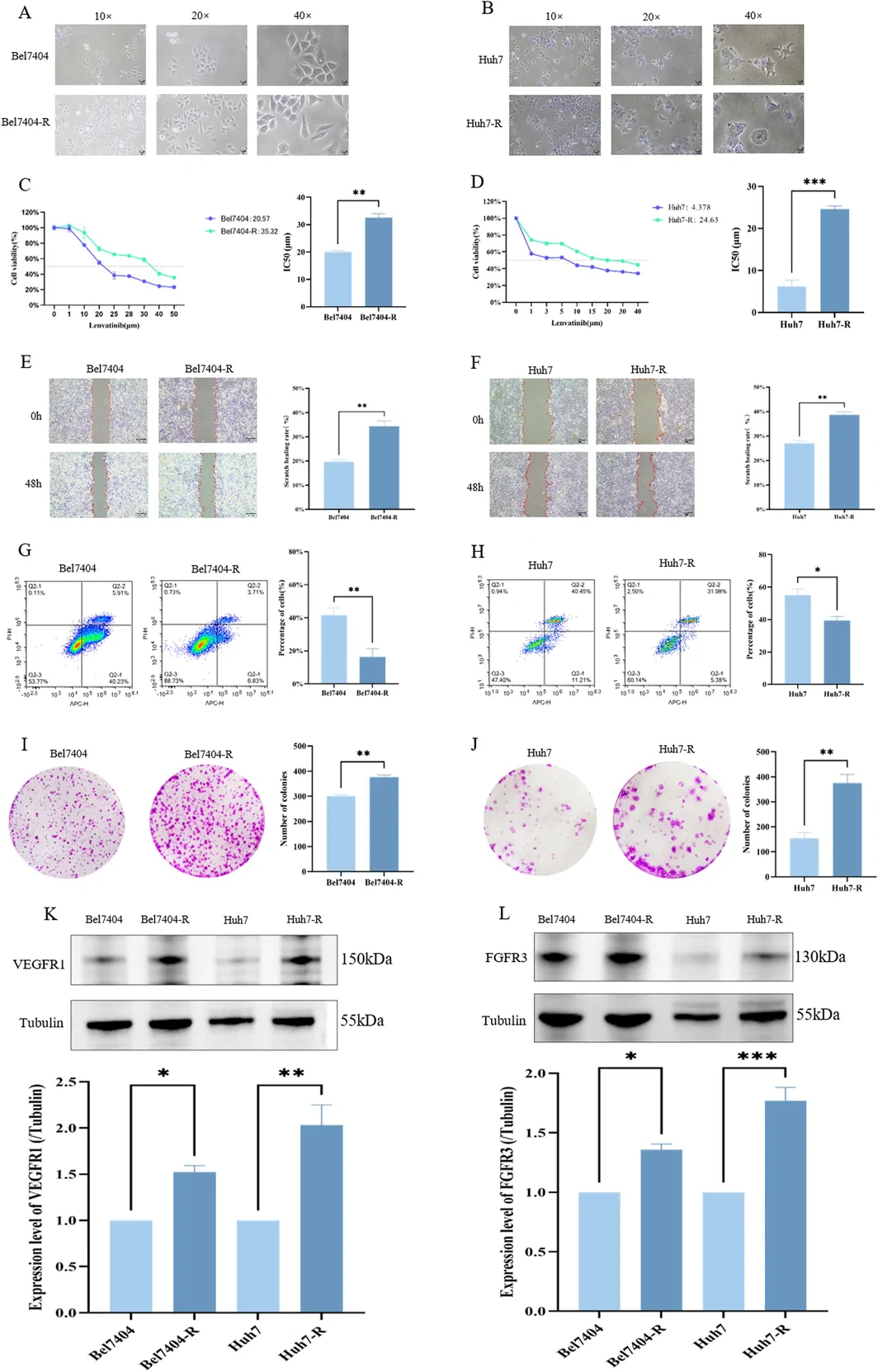
Construction of lenvatinib‐resistant HCC cell lines and the expression of VEGFR1 and FGFR3 in normal and drug‐resistant HCC cells. (A) Cell morphology of Bel7404 and Bel7404‐R under drug screening conditions. (B) Cell morphology of Huh7 and Huh7‐R under drug screening conditions. (C) IC_50_ of Bel7404 and Bel7404‐R under the treatment of lenvatinib. (D) IC_50_ of Huh7 and Huh7‐R under the treatment of lenvatinib. (E) The scratch healing rates of Bel7404 and Bel7404‐R under the treatment of lenvatinib. (F) The scratch healing rate of Huh7 and Huh7‐R under the treatment of lenvatinib. (G) The apoptosis rate of Bel7404 and Bel7404‐R under the treatment of lenvatinib. Apoptosis was quantified by flow cytometry using Annexin V‐APC/PI double staining. The total apoptotic rate (early + late) is shown as mean ± SD from three independent experiments. (H) The apoptosis rates of Huh7 and Huh7‐R under the treatment of lenvatinib. Apoptosis was quantified by flow cytometry using Annexin V‐APC/PI double staining. The total apoptotic rate (early + late) is shown as mean ± SD from three independent experiments. (I) The cell clone formation ability of Bel7404 and Bel7404‐R under the treatment of lenvatinib. (J) The cell clone formation ability of Huh7 and Huh7‐R under the treatment of lenvatinib. (K) The expression of VEGFR1 in normal and drug‐resistant HCC cells, the bar chart on the below shows the statistics of the scanning grey values of the protein bands. Error bars represent SD of normalized band intensities from three independent experiments, where the parental cell group was set to 1. (L) The expression of FGFR3 in normal and drug‐resistant HCC cells. Error bars represent SD of normalized band intensities from three independent experiments, where the parental cell group was set to 1. Statistical analysis was performed as described in Methods. *p < 0.05; **p < 0.01; ***p < 0.001; ^ns^ *p* > 0.05.

### 3.3 The Emergence of Lenvatinib Resistance Is Associated With Inhibition of the Ferroptosis Pathway in HCC Cells

The ferrous ion levels in the drug‐resistant cell lines (Bel7404‐R and Huh7‐R) were significantly lower than those in their parental cells (Bel7404 and Huh7) (*p* < 0.05) (Figure 3A). A significant decrease in mean fluorescence intensity (MFI), suggesting a reduction in reactive oxygen species (ROS) levels, was demonstrated by flow cytometry in the drug‐resistant cells. This was evident in both the Bel7404‐R (*p* < 0.05) and Huh7‐R (*p* < 0.01) groups (Figure 3B). Lipid peroxidation detection further confirmed that the degree of lipid peroxidation in drug‐resistant cells was significantly lower than that in the parental cells (*p* < 0.001). The GSH/GSSG assay indicated that GSH content in drug‐resistant cells was significantly increased (*p* < 0.01) (Figure 3C,D). Immunofluorescence analysis showed that the fluorescence intensities of the ferroptosis‐promoting factors ACSL4 and TFR1 in drug‐resistant cells were weakened, while the fluorescence intensities of inhibitory factors FTH1 and SLC7A11 were enhanced. The changes in the expression profile of this protein showed a consistent trend in intergroup comparisons between Bel7404‐R and Bel7404, Huh7‐R, and Huh7 (Figure 3E–H), suggesting that lenvatinib resistance is related to resistance of the cellular ferroptosis pathway.

**FIGURE 3:**
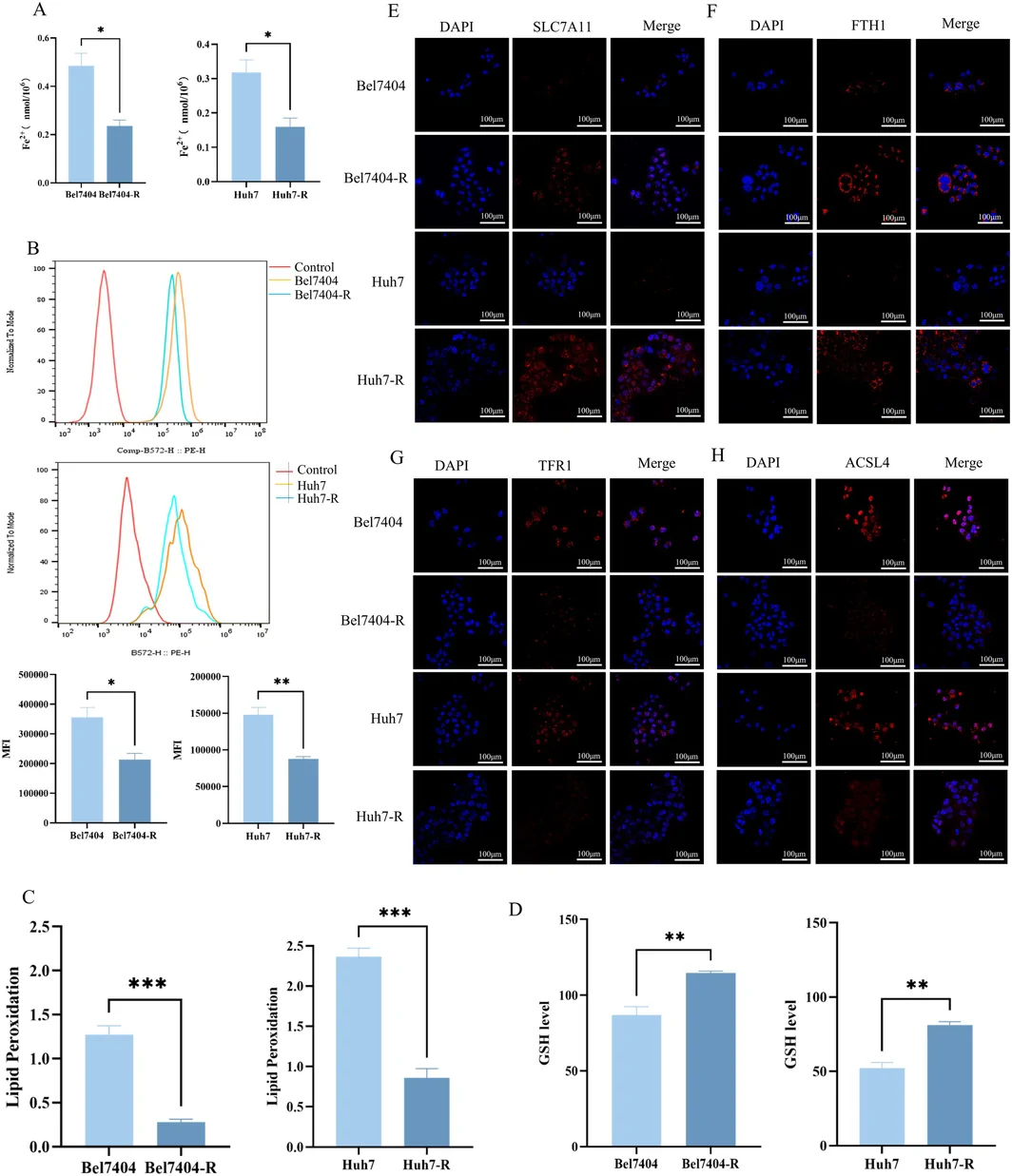
Correlation between lenvatinib resistance and ferroptosis‐related indicators. (A) Detect the levels of ferrous ions in Bel7404, Bel7404‐R, Huh7, and Huh7‐R cells; (B) Flow cytometry was used to detect the reactive oxygen species levels in Bel7404, Bel7404‐R, Huh7, and Huh7‐R cells; the below bar chart is the statistical analysis of reactive oxygen species levels. (C) Detect the lipid peroxidation levels of Bel7404, Bel7404‐R, Huh7, and Huh7‐R cells; (D) Detect the GSH (glutathione) levels in Bel7404, Bel7404‐R, Huh7, and Huh7‐R cells; (E, F) Fluorescence intensity of SLC7A11 and FTH1 in drug‐resistant cells HCC and parental cells; (G, H) Fluorescence intensity of TFR1 and ACSL4 in drug‐resistant HCC cells and parental cells; the length of the scale is 100 μm. ACSL4, long‐chain acyl‐CoA synthetase 4; FTH1, ferritin heavy chain; SLC7A11, member 11 of the solute carrier family 7; TFR1, transferrin receptor 1. Statistical analysis was performed as described in Methods. *p < 0.05; **p < 0.01; ***p < 0.001; ^ns^ *p* > 0.05.

### 3.4 HuR Was Upregulated in Drug‐Resistant Cell Lines, and the Drug‐Resistant Phenotype Decreased After Interfering With HuR

We successfully established drug‐resistant liver cancer cell lines with stable HuR knockdown to investigate the role of upregulated HuR expression. Through literature review and analysis of bioinformatics databases, it was found that HuR was upregulated in HCC cells. Therefore, we detected the expression level of HuR in normal liver cells (THLE) and HCC cell lines (PLC, HLE, Huh7, Bel7404) using western blotting, as shown in Figure 4A. The expression level of HuR in THLE cells was lower than that in PLC, HLE, Huh7, and Bel7404 cells, and this difference was statistically significant (*p* < 0.05). We further explored whether drug‐resistant HCC cells exert resistance to lenvatinib by upregulating HuR levels. First, differences in HuR at the transcriptional level between drug‐resistant and parental HCC cells were detected by qRT‐PCR. The results showed that the transcriptional levels of HuR in Bel7404‐R and Huh7‐R were higher than those in the parental HCC cell lines Bel7404 and Huh7 (Figure 4B); Further, through cell protein extraction and Western blotting assays, the expression level of HuR in drug‐resistant and parental HCC cells was detected. The results showed that the expression levels of HuR in Bel7404‐R and Huh7‐R cells were significantly higher than those in Bel7404 and Huh7 cells (*p* < 0.05) (Figure 4C). We also detected the cellular localization and expression of HuR in HCC cells and drug‐resistant cells using cellular immunofluorescence experiments. As shown in Figure 4D, HuR was mainly located in the cell nucleus. It can be known from the pictures that the fluorescence intensity of Bel7404‐R cells is higher than that of Bel7404 cells. Moreover, the fluorescence intensity of Huh7‐R cells was higher than that of Huh7 cells, indicating that the expression of HuR in drug‐resistant cells was higher than that in parental liver cancer cells. These results suggest that HuR may be associated with drug resistance in liver cancer. Lentiviral vectors with HuR knockdown were transfected into Bel7404‐R and Huh7‐R cells, and an empty control group was set up simultaneously. The transfection efficiency of the cells was observed and photographed under a fluorescence microscope (Figure 4E,G). By comparing the images of the cells under white light and green fluorescence, we observed that the transfection efficiency of the cells reached 90%. The results of the Western blotting experiment showed that there was no statistically significant difference in HuR expression between the Bel7404‐R and Bel7404‐R‐NC groups (Figure 4F). However, compared with the Bel7404‐R group and the negative vector group (Bel7404‐R‐NC), the expression level of HuR in the HuR knockdown group (Bel7404‐R‐shHuR) was significantly decreased (*p* < 0.001), and the results also indicated that the expression of HuR in the Bel7404‐R group was higher than that in the Bel7404 group (*p* < 0.05), which was consistent with previous experimental results. As shown in Figure 4H, there was no statistically significant difference in HuR expression between Huh7‐R and Huh7‐R‐NC groups. However, compared to the Huh7‐R group and Huh7‐R‐NC group, HuR the expression levels of HuR protein in the Huh7‐R‐shHUR group were significantly downregulated (*p* < 0.01). Immunofluorescence results showed that the fluorescence intensities in both Bel7404‐R and Bel7404‐R‐NC groups were stronger than those in Bel7404 and Bel7404‐R‐shHuR groups. The fluorescence intensities of the Huh7‐R and Huh7‐R‐NC groups were stronger than those of the Huh7 and Huh7‐R‐shHuR groups (Figure 4I). Our experimental results showed that a liver cancer drug‐resistant cell line with stable knockdown of HuR was successfully constructed.

**FIGURE 4:**
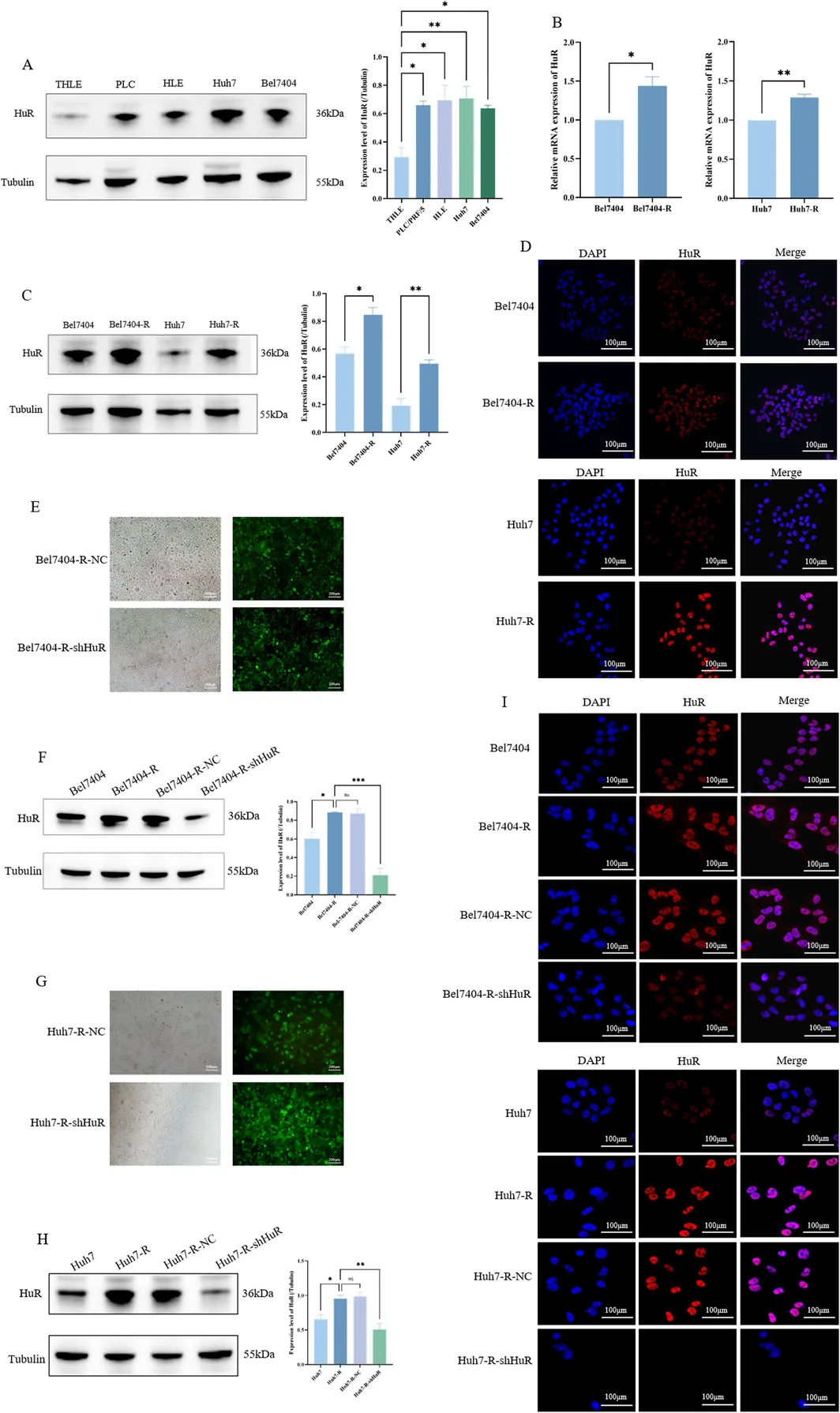
Upregulated expression of HuR positive related to lenvatinib resistance and established stable HuR knockdown in drug‐resistant HCC cell lines. (A) The expression level of HuR in normal hepatocytes (THLE) and HCC cells (PLC, HLE, Huh7 and Bel7404) was detected by Western blotting, the bar chart on the right shows the statistics of the scanning grey values of the protein bands. (B) The transcription levels of HuR in drug‐resistant cells and parental cells were detected by qRT‐PCR assay. (C) The Western blotting was used to detect the expression level of HuR in Bel7404, Bel7404‐R, Huh7 and Huh7‐R, the bar chart on the right shows the statistics of the scanning grey values of the protein bands. (D) The expression and localization of HuR in drug‐resistant HCC cells and parental cells were detected by immunofluorescence; (E) The transfection efficiency of the Bel7404‐R‐shHuR was observed by fluorescence microscopy. (F) Western blotting was used to detect the expression level of HuR in Bel7404‐R cells after knockdown of HuR (Bel7404‐R‐shHuR), the bar chart on the right shows the statistics of the scanning grey values of the protein bands. (G) The transfection efficiency of the Huh7‐R‐shHuR by fluorescence microscopy. (H) Western blotting was used to detect the expression level of HuR in Huh7‐R cells after knockdown of HuR (Huh7‐R‐shHuR), the bar chart on the right shows the statistics of the scanning grey values of the protein bands. (I) The localization and expression of HuR in Bel7404‐R‐shHuR and Huh7‐R‐shHuR cells were observed by immunofluorescence assay, the length of the scale is 100 μm. Statistical analysis was performed as described in Methods. *p < 0.05; **p < 0.01; ***p < 0.001; ^ns^ *p* > 0.05.

### 3.5 Knockdown the Expression of HuR Could Inhibit the Malignant Behaviour of HCC Cells

It was found through the CCK8 experiment that the IC_50_ values of Bel7404‐R and Huh7‐R were significantly increased by 1.8 times (17.46 vs. 32.10 μM, *p* < 0.001) and 22 times (1.281 vs. 22.68 μM), respectively, compared with their parental cells (Bel7404 and Huh7) (*p* < 0.001) (Figure 5A). After HuR knockdown, the drug sensitivity of Bel7404‐R and Huh7‐R cells was significantly restored. The IC_50_ values of Bel7404‐R‐shHuR and Huh7‐R‐shHuR decreased to 16.99 μM and 5.276 μM, respectively, which were 1.9 times (*p* < 0.001) and 3.9 times (*p* < 0.001) lower than those of the corresponding drug‐resistant groups. The scratch assay showed that the healing rate of the drug‐resistant group (Bel7404‐R/Huh7‐R) was significantly higher than that of the HuR knockdown group (*p* < 0.01/*p* < 0.001) and parental cells (*p* < 0.01/*p* < 0.001) (Figure 5B). Under lenvatinib treatment, the clone formation ability of drug‐resistant cells was significantly stronger than that of the parental cells (Bel7404‐R vs. Bel7404, *p* < 0.001; Huh7‐R vs. Huh7, *p* < 0.05), while the clone formation ability of drug‐resistant cells was significantly weakened after knockdown of HuR (Bel7404‐R‐shHuR vs. Bel7404‐R, *p* < 0.001; Huh7‐R‐shHuR vs. Huh7‐R, *p* < 0.001) (Figure 5C). These results indicate that HuR is involved in lenvatinib resistance by enhancing the proliferation and migration abilities of HCC cells, and that the HuR expression level is positively correlated with drug resistance.

**FIGURE 5:**
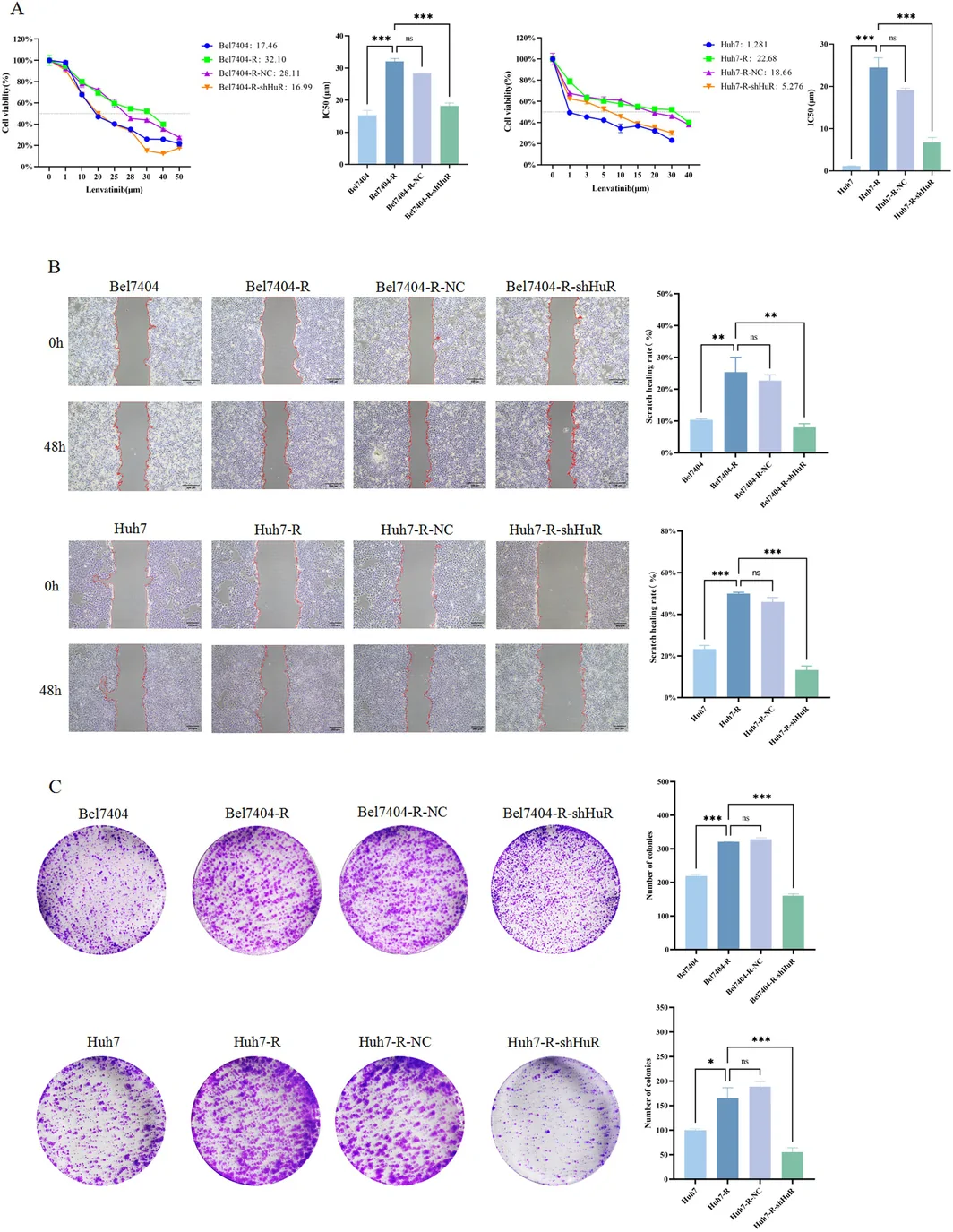
The effect of HuR in the malignant behaviours of HCC cells. (A) CCK8 was applied to detect the IC_50_ of Bel7404‐R‐shHuR and Huh7‐R‐shHuR. (B) Scratch healing of Bel7404‐R‐shHuR and Huh7‐R‐shHuR cells at 0 and 48 h. (C) Plate cloning assay was used to detect the proliferation ability of Bel7404‐R‐shHuR and Huh7‐R‐shHuR cells. Statistical analysis was performed as described in Methods. *p < 0.05; **p < 0.01; ***p < 0.001; ^ns^ *p* > 0.05.

### 3.6 Knockdown the Expression of HuR Could Increase Ferroptosis in Drug‐Resistant HCC Cells

By detecting the indicators related to ferroptosis, it was found that the Fe^2+^ content in the drug‐resistant cells, Bel7404‐R and Huh7‐R, was significantly lower than that in their parental cells (*p* < 0.05), while the Fe^2+^ level significantly rebounded after knockdown of HuR expression (*p* < 0.01) (Figure 6A). The ROS and lipid peroxidation levels of drug‐resistant HCC cells were significantly lower than those of the corresponding parental cells and the HuR knockout group (*p* < 0.05, *p* < 0.001) (Figure 6B,C), while the GSH level was significantly higher than that of the parental cells and HuR knockout group (*p* < 0.01 and *p* < 0.05) (Figure 6D). This trend was consistent in both Bel7404 and Huh7 cell lines, suggesting that HuR might inhibit ROS accumulation and lipid peroxidation by upregulating GSH, thereby reducing the ferroptosis sensitivity of drug‐resistant cells. As an important RNA‐binding protein, HuR enhances the stability of specific mRNAs and prevents their degradation by binding to their 3′‐untranslated region in the cytoplasm. Therefore, the cytoplasmic expression level of HuR is an important factor in its function. By extracting the proteins from the nuclei and cytoplasm of drug‐resistant cells and parent cells, and through Western blotting experiments, we determined the expression levels of HuR in the nuclei, cytoplasm, and total proteins. The experimental results indicated that the expression level of HuR in the cytoplasm of drug‐resistant HCC cells was higher than that in parental cells (*p* < 0.001, *p* < 0.01) (Figure 6E). As shown in Figure 6F, the expression level of GPX4 in Bel7404‐R cells was higher than that in Bel7404 cells (*p* < 0.001), and the expression of GPX4 in Huh7‐R cells was also higher than that in Huh7 cells (*p* < 0.05); Further, the changes of GPX4 at the transcriptional level in Bel7404‐R, Bel7404, Huh7‐R and Huh7 cells were detected by qRT‐PCR. The results showed that the level of GPX4 mRNA in Bel7404‐R cells was higher than that in Bel7404 cells (*p* < 0.001). Moreover, the level of GPX4 mRNA in Huh7‐R cells was higher than that in Huh7 cells (*p* < 0.05) (Figure 6G). These results indicated that the expression of GPX4 is related to lenvatinib resistance in HCC.

**FIGURE 6:**
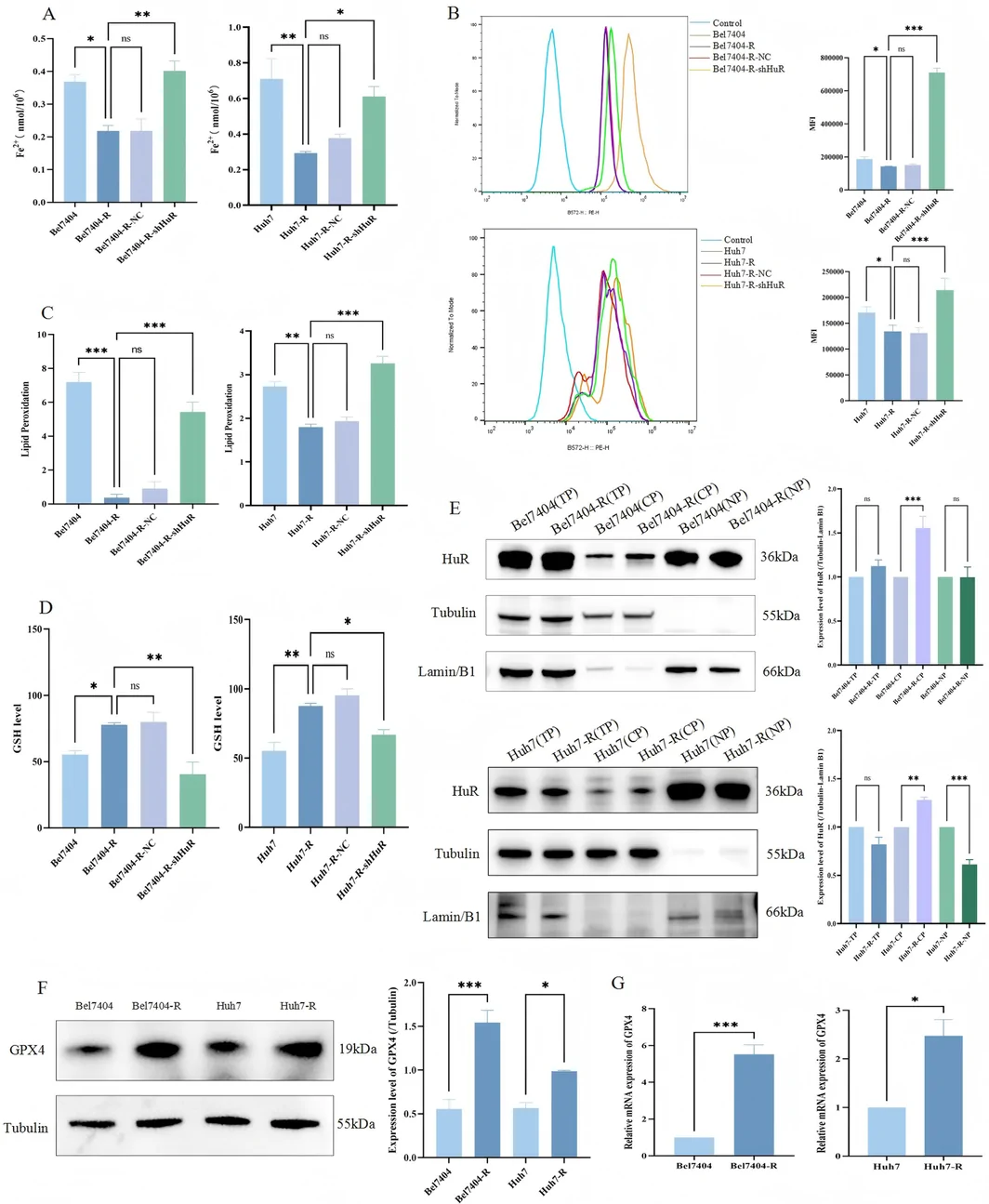
The influence of HuR on the intracellular ferroptosis‐related indicators in HCC cells. (A) The change of ferrous ions in Bel7404‐R and Huh7‐R cells while interfered with the expression of HuR (shHuR). (B) The change in the content of reactive oxygen species in Bel7404‐R and Huh7‐R cells while interfered with the expression of HuR (shHuR). (C) The change of lipid peroxidation in Bel7404‐R and Huh7‐R cells while interfered with the expression of HuR (shHuR). (D) The change of GSH in Bel7404‐R and Huh7‐R cells while interfered with the expression of HuR (shHuR). (E) The expression of HuR in total protein (TP), cytoplasmic protein (CP), and nuclear protein (NP) in Bel7404, Bel7404‐R, Huh7, and Huh7‐R, the bar chart on the right shows the statistics of the scanning grey values of the protein bands. (F) The expression of GPX4 in Bel7404, Bel7404‐R, Huh7, and Huh7‐R cells; the bar chart on the right shows the statistics of the scanning grey values of the protein bands. (G) Transcriptional levels of GPX4 mRNA in Bel7404, Bel7404‐R, Huh7, and Huh7‐R cells. Statistical analysis was performed as described in Methods. *p < 0.05; **p < 0.01; ***p < 0.001; ^ns^ *p* > 0.05.

### 3.7 The Expression Level of GPX4 in Drug‐Resistant HCC Cells Was Significantly Increased and Positively Correlated With the Expression of HuR

After knockdown, HuR expression downregulated GPX4 expression in drug‐resistant HCC cells. The expression of GPX4 in drug‐resistant HCC cells and parental cells was detected using immunofluorescence. The results indicated that the fluorescence intensity of GPX4 in Bel7404‐R and Huh7‐R cells was significantly higher than that in Bel7404 and Huh7 cells (Figure 7A). Furthermore, Western blotting was conducted to detect whether there was a difference in the expression of GPX4 between the drug‐resistant cell lines after knockdown of HuR (Bel7404‐R‐shHuR, Huh7‐R‐shHuR) and drug‐resistant HCC cells. These results indicated that there was no significant difference in the expression of GPX4 between Bel7404‐R and Bel7404‐R‐NC. The expression of GPX4 in Bel7404‐R‐shHuR and Bel7404 cells was significantly lower than that in Bel7404‐R cells, and both were statistically significant (*p* < 0.01). The expression of GPX4 in Huh7‐R cells was not significantly different from that in Huh7‐R‐NC cells, whereas the expression of GPX4 in HuH7‐R‐shHuR and Huh7 cells was significantly lower than that in Huh7‐R cells (*p* < 0.01) (Figure 7B). Immunofluorescence experiments also showed that the fluorescence intensity of GPX4 in Bel7404‐R‐shHuR and Huh7‐R‐shHuR cells was significantly lower than in Bel7404‐R and Huh7‐R cells (Figure 7C). The results showed that the expression of GPX4 was related to drug resistance in liver cancer and changed along with the expression of HuR.

**FIGURE 7:**
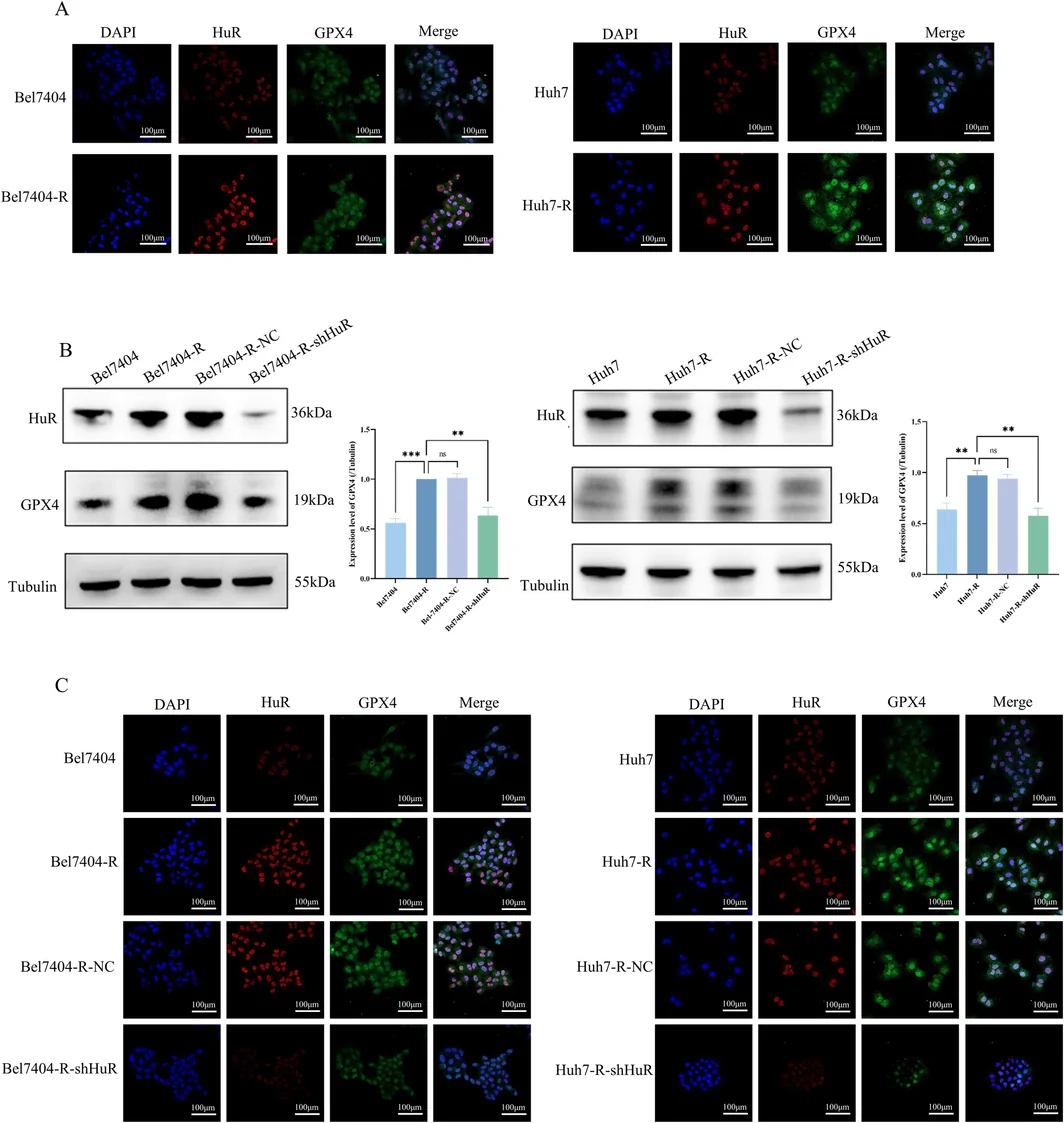
The influence of HuR on the expression of GPX4 in HCC cells. (A) The expression and location of GPX4 in Bel7404, Bel7404‐R, Huh7, and Huh7‐R cells was observed by immunofluorescence assay. (B) Western blotting was conducted to detect the expression of HuR and GPX4 in Bel7404‐R and Huh7‐R cells while interfered with the expression of HuR (shHuR); the bar chart on the right shows the statistics of the scanning grey values of the protein bands. (C) Immunofluorescence was used to detect the expression and location of HuR and GPX4 in Bel7404‐R and Huh7‐R cells while interfered with the expression of HuR (shHuR). The length of the scale is 100 μm. Statistical analysis was performed as described in Methods. **p < 0.01; ***p < 0.001; ^ns^ *p* > 0.05.

### 3.8 Construct a Cell Line Stably Transfected With shHuR‐GPX4

Lentiviral vectors overexpressing GPX4 were transfected into shHuR cells and verified by Western blotting and immunofluorescence. As shown in Figure 8A,B, the expression of GPX4 in Bel7404‐R was not significantly different from that in Bel7404‐R‐NC and Bel7404‐R‐shHuR‐GPX4. The expression of GPX4 in Bel7404‐R‐shHuR and Bel7404 cells was significantly lower than that in Bel7404‐R cells, and both were statistically significant (*p* < 0.05). Moreover, the expression of GPX4 in Bel7404‐R‐shHuR‐GPX4 cells was higher than that in Bel7404‐R‐shHuR cells (*p* < 0.001); The expression of GPX4 in Huh7‐R showed no significant difference from that in HuH7‐R‐NC and HuH7‐R‐shHuR‐GPX4, while the expression of GPX4 in HuH7‐R‐shHuR and Huh7 cells was significantly lower than that in Huh7‐R cells (*p* < 0.05). Moreover, the expression of GPX4 in HuH7‐R‐shHuR‐GPX4 cells was higher than that in Huh7‐R‐shHuR cells (*p* < 0.05), indicating that the GPX4 overexpression cell line had been successfully constructed. In addition, the immunofluorescence experiment fully demonstrated this point and was mutually verified by the Western blotting experiment, as shown in Figure 8C,D.

**FIGURE 8:**
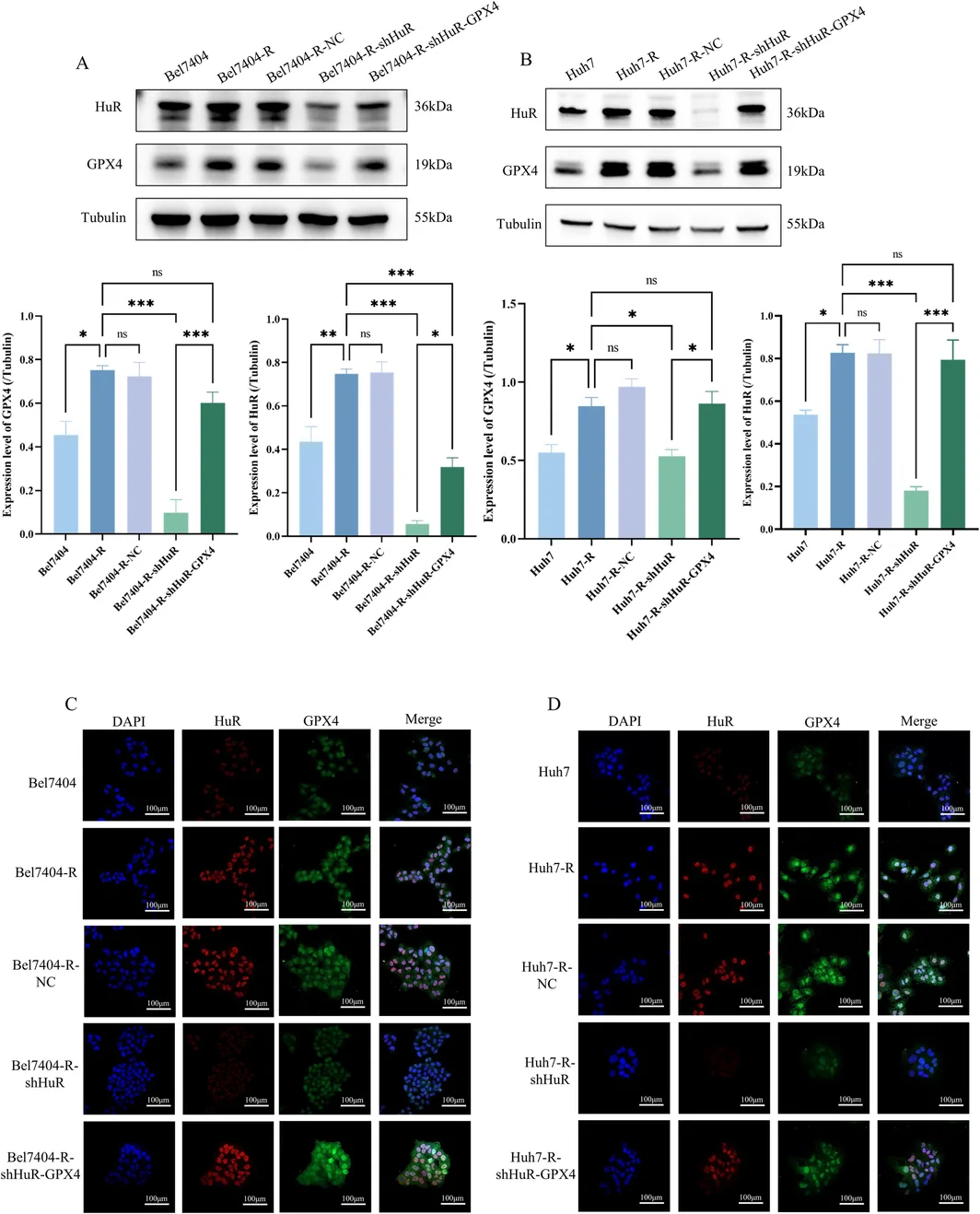
Construct cell lines that stably overexpress GPX4 in lenvatinib resistant HCC‐shHuR cells (shHuR‐GPX4). (A) The Western blotting was used to detect the expression of HuR and GPX4 in Bel7404, Bel7404‐R, Bel7404‐R‐NC, Bel7404‐R‐shHuR, and Bel7404‐R‐shHuR‐GPX4; the bar chart below shows the statistics of the scanning grey values of the protein bands. (B) The expression of HuR and GPX4 in Huh7, Huh7‐R, Huh7‐R‐NC, Huh7‐R‐shHuR, and HuH7‐R‐shHuR‐GPX4 was detected by Western blotting, the bar chart below shows the statistics of the scanning grey values of the protein bands. (C) Immunofluorescence assay was used to detect the expression and location of HuR and GPX4 in Bel7404, Bel7404‐R, Bel7404‐R‐NC, Bel7404‐R‐shHuR, and Bel7404‐R‐shHuR‐GPX4. (D) The expression and location of HuR and GPX4 in Huh7, Huh7‐R, HUH7‐R‐NC, HuH7‐R‐shHuR, and HuH7‐R‐shHuR‐GPX4 was detected by immunofluorescence assay, the length of the scale is 100 μm. Statistical analysis was performed as described in Methods. *p < 0.05; **p < 0.01; ***p < 0.001; ^ns^ *p* > 0.05.

### 3.9 Knockdown HuR and Overexpression of GPX4 in Drug‐Resistant HCC Cells, the Malignant Behaviour of the Cells Was Somewhat Restored

This study systematically evaluated the mechanism of HuR and GPX4 proteins in the resistance of HCC cells to lenvatinib using multiple experimental methods. The CCK8 experimental results showed that the IC_50_ values (32.81 and 32.81 μM, respectively) of the Bel7404‐R and Huh7‐R were significantly increased by 3.6 times and 16.7 times respectively compared with their parental cells, Bel7404 and Huh‐7 (15.71 μM and 1.921 μM, respectively) (*p* < 0.001). When the expression of HuR was interfered with, the IC_50_ values of Bel7404‐R‐shHuR and Huh7‐R‐shHuR decreased to 10.78 μM and 12.19 μM, respectively, which were 3 times and 1.8 times lower than those of the drug‐resistant HCC cell line (*p* < 0.001), while the overexpression of GPX4 could cause the IC_50_ to rebound to 33.73 and 28.60 μM. It increased by 2.9 times and 2.7 times compared to the shHuR group (*p* < 0.001 and *p* < 0.01, respectively). It is worth noting that there was no statistically significant difference in IC_50_ between the blank control (NC) group and the GPX4 overexpression group and the drug‐resistant HCC cells, confirming the core role of HuR in regulating drug resistance and the compensatory effect mediated by GPX4 (Figure 9A).

**FIGURE 9:**
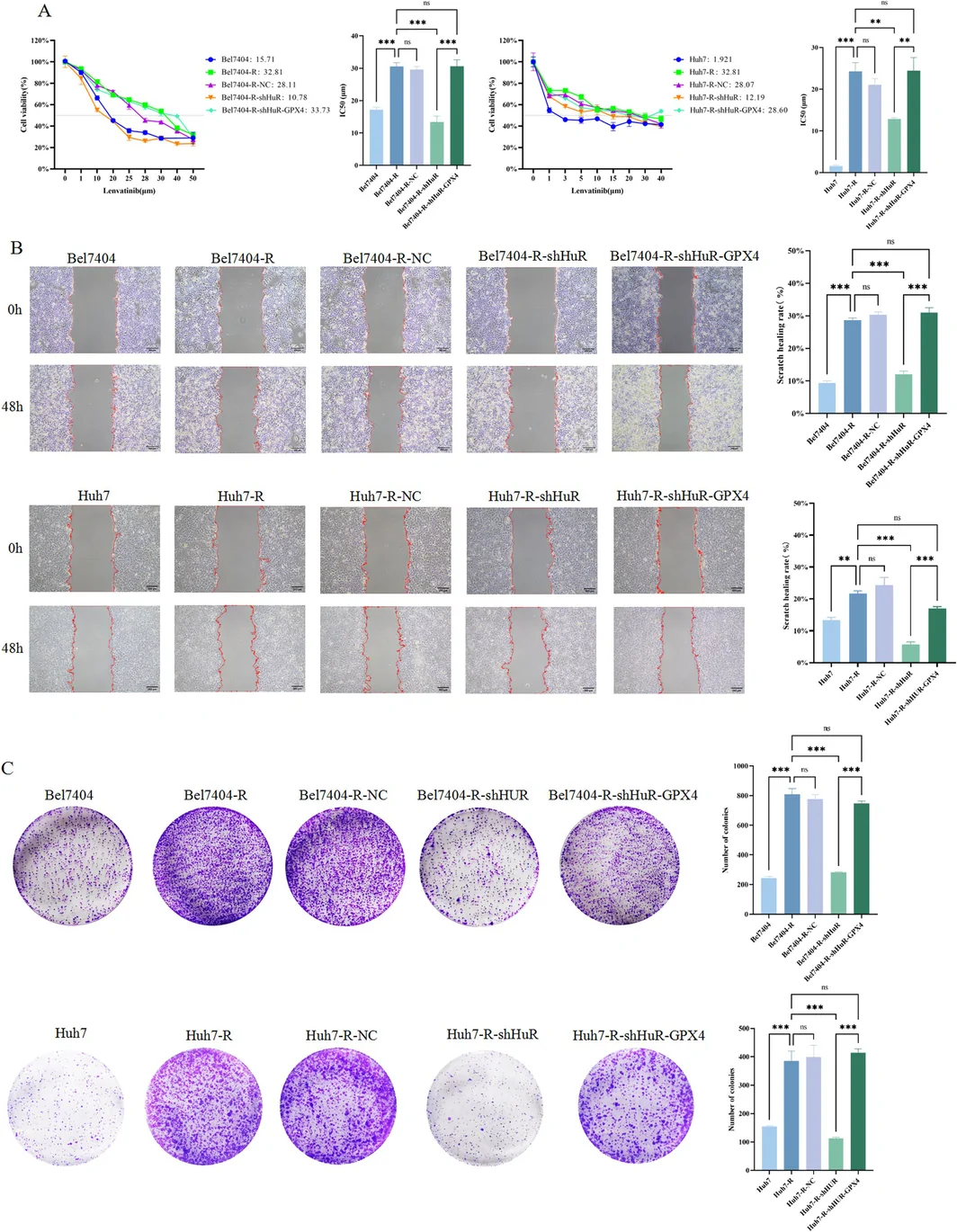
The effect of HuR‐GPX4 axis in malignant behaviours of HCC cells. (A) CCK8 was used to detect the IC_50_ of lenvatinib in Bel7404, Bel7404‐R, Bel7404‐R‐NC, Bel7404‐R‐shHuR, and Bel7404‐R‐shHuR‐GPX4 cells; Huh7, Huh7‐R, Huh7‐R‐NC, Huh7‐R‐shHuR, and Huh7‐R‐shHuR; the bar chart on the right is the statistical analysis of IC_50_. (B) Scratch healing of Bel7404, Bel7404‐R, Bel7404‐R‐NC, Bel7404‐R‐shHuR, and Bel7404‐R‐shHuR‐GPX4 cells; Huh7, Huh7‐R, Huh7‐R‐NC, HuH7‐R‐shHuR, and HuH7‐R‐shHuR‐GPX4 cells at 0 and 48 h; the bar chart on the right shows the statistical analysis of the repair distance. (C) Plate cloning assay was used to detect the proliferation ability of Bel7404, Bel7404‐R, Bel7404‐R‐NC, Bel7404‐R‐shHuR, and Bel7404‐R‐shHuR‐GPX4 cells; Huh7, Huh7‐R, Huh7‐R‐NC, HuH7‐R‐shHuR, and HuH7‐R‐shHuR‐GPX4 cells; the bar chart on the right is a statistical analysis of the number of cell clones. Statistical analysis was performed as described in Methods. **p < 0.01; ***p < 0.001; ^ns^ *p* > 0.05.

Functional experiments further verified the above mechanism. In the scratch experiment, the healing rates of Bel7404‐R and Huh7‐R were significantly higher than those of the parental cells (*p* < 0.001), but shHuR treatment significantly decreased their healing rates (*p* < 0.001), while overexpression of GPX4 partially restored their migration ability (*p* < 0.001) (Figure 9B). Plate cloning experiments under lenvatinib treatment showed that the clone formation ability of drug‐resistant strains was enhanced compared with that of parental cells (*p* < 0.001). shHuR significantly inhibited clone formation (*p* < 0.001), while overexpression of GPX4 restored the clone number to a level close to that of drug‐resistant strains (*p* < 0.001) (Figure 9C). The results of the two groups of cell lines were highly consistent, jointly demonstrating that HuR affects the drug sensitivity, migration, and proliferation ability of HCC cells by regulating the expression of GPX4 and clarifying the key molecular mechanism of lenvatinib resistance.

### 3.10 Knockdown HuR and Overexpression of GPX4 in Drug‐Resistant HCC Cells Was Able to Inhibit Ferroptosis

In this study, by detecting iron homeostasis and oxidative stress‐related indicators, we elucidated the molecular mechanism by which the HuR‐GPX4 axis regulates lenvatinib resistance in HCC cells. Iron metabolism detection showed that there was no significant difference in the Fe^2+^ content between Bel7404‐R and Huh7‐R and the negative control (NC) and GPX4 overexpression groups (shHuR‐GPX4). However, the Fe^2+^ levels of the parental cells (Bel7404, Huh7) and HuR interference group (shHuR) were significantly higher than those of the Bel7404‐R and Huh7‐R cells (*p* < 0.05), whereas the overexpression of GPX4 reduced the Fe^2+^ content of shHuR cells back to the level of drug‐resistant HCC cells (*p* < 0.05) (Figure 10A). Correspondingly, flow cytometry analysis revealed that the reactive oxygen species (ROS) levels of Bel7404‐R and Huh7‐R were significantly lower than those of the parent strain and the shHuR group (*p* < 0.001), whereas the overexpression of GPX4 reversed the increase in ROS caused by shHuR (*p* < 0.001) (Figure 10B). Further analysis of the lipid peroxidation marker malondialdehyde (MDA) showed that the MDA levels in drug‐resistant HCC cells were lower than those in the parental strain and the shHuR group (*p* < 0.05), and the overexpression of GPX4 significantly inhibited the accumulation of MDA in the shHuR group (*p* < 0.05) (Figure 10C). The results of glutathione (GSH) detection showed a reverse trend: The GSH content of the drug‐resistant HCC cells was significantly higher than that of the parental cells and the shHuR group (*p* < 0.01), while the overexpression of GPX4 partially restored GSH depletion in the shHuR group (*p* < 0.01) (Figure 10D). Transmission electron microscopy revealed that under lenvatinib treatment, Bel7404 cells presented a characteristic mitochondrial damage phenotype, the density of the inner mitochondrial membrane was significantly increased, the arrangement of crista structures was disordered, and some areas had completely disappeared. Notably, the drug‐resistant cell line Bel7404‐R and its negative control Bel7404‐R‐NC showed a trend toward mitochondrial ultrastructural normalization. Further studies have shown that in Bel7404‐R‐shHuR cells induced by HuR gene silencing, ferroptose‐related pathological changes occurred again in mitochondria: mitochondrial morphology disappeared, cell membrane and nucleus were damaged, and intracellular lipid droplets increased; After the intervention of GPX4 overexpression, the degree of mitochondrial damage in this group of cells was alleviated compared with the Bel7404‐R‐shHuR group (Figure 10E). This indicates that HuR maintains the REDOX homeostasis of drug‐resistant HCC cells and ultimately enhances their tolerance to lenvatinib by regulating the expression of GPX4 and affecting the key factor (Fe^2+^/ROS/MDA/GSH/Mitochondrial damage) of the ferroptosia‐related pathway. This discovery, together with the previous results of CCK8, scratch healing, and clone formation experiments, constructs the core mechanism of HuR‐GPX4‐mediated ferroptosis resistance in drug resistance of liver cancer.

**FIGURE 10:**
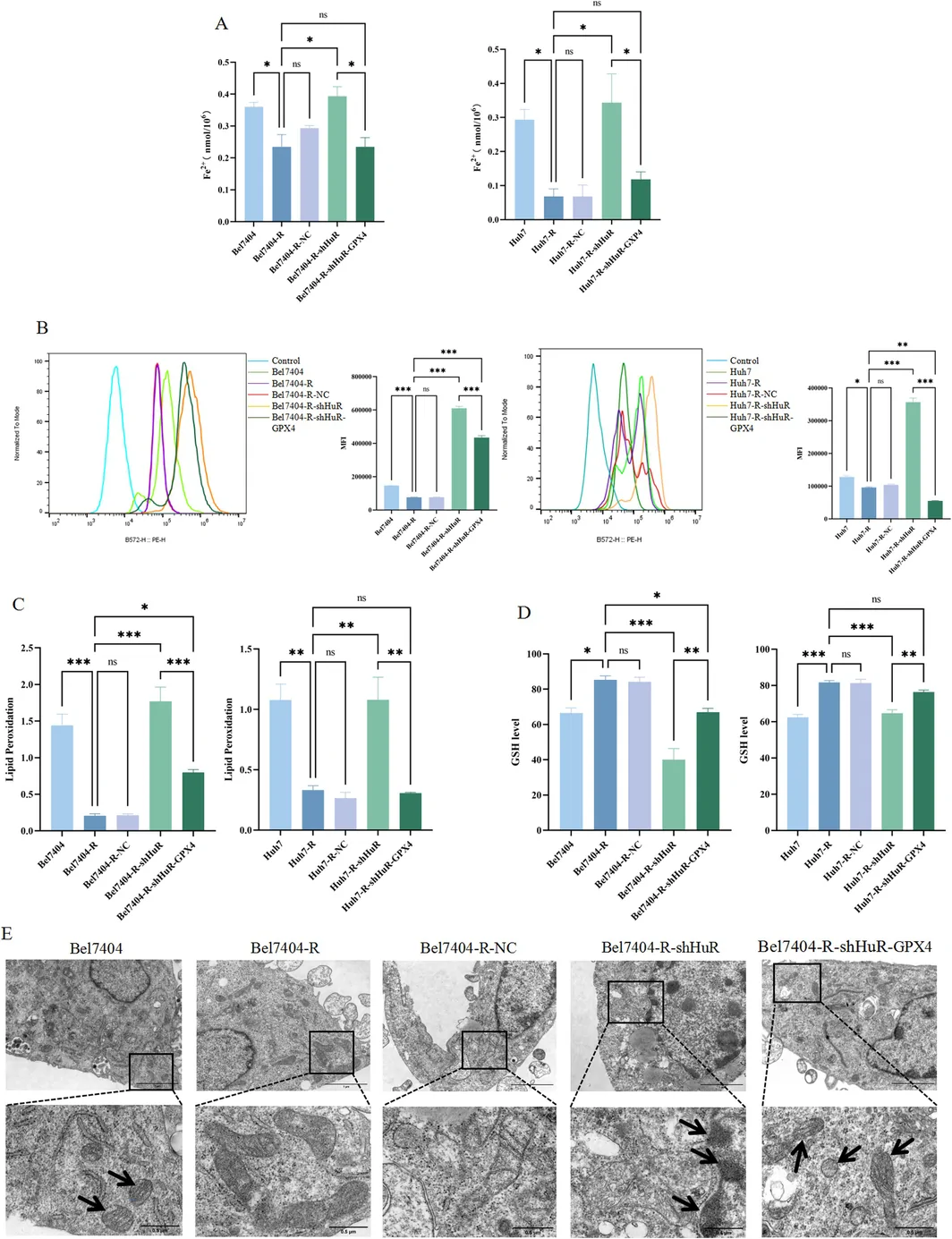
The influence of HuR‐GPX4 axis on the ferroptosis‐related indicators in HCC cells. (A) The contents of ferrous ions (Fe^2+^) in Bel7404, Bel7404‐R, Bel7404‐R‐NC, Bel7404‐R‐shHuR, and Bel7404‐R‐shHuR‐GPX4 cells; Huh7, Huh7‐R, Huh7‐R‐NC, HuH7‐R‐shHuR, and HuH7‐R‐shHuR‐GPX4 cells. (B) The content of reactive oxygen species in Bel7404, Bel7404‐R, Bel7404‐R‐NC, Bel7404‐R‐shHuR, and Bel7404‐R‐shHuR‐GPX4 cells; Huh7, Huh7‐R, Huh7‐R‐NC, HuH7‐R‐shHuR, and HuH7‐R‐shHuR‐GPX4 cells; the bar chart on the right shows the statistical analysis of ROS content (MFI). (C) The contents of lipid peroxidation in Bel7404, Bel7404‐R, Bel7404‐R‐NC, Bel7404‐R‐shHuR, and Bel7404‐R‐shHuR‐GPX4 cells; Huh7, Huh7‐R, Huh7‐R‐NC, HuH7‐R‐shHuR, and HuH7‐R‐shHuR‐GPX4 cells. (D) The contents of GSH (glutathione) in Bel7404, Bel7404‐R, Bel7404‐R‐NC, Bel7404‐R‐shHuR, and Bel7404‐R‐shHuR‐GPX4 cells; Huh7, Huh7‐R, Huh7‐R‐NC, HuH7‐R‐shHuR, and HuH7‐R‐shHuR‐GPX4 cells. (E) The ferroptosome of Bel7404, Bel7404‐R, Bel7404‐R‐NC, Bel7404‐R‐shHuR, and Bel7404‐R‐shHuR‐GPX4 cells was observed by transmission electron microscopy after treated with lenvatinib (20 μM) for 48 h. Statistical analysis was performed as described in Methods. *p < 0.05; **p < 0.01; ***p < 0.001; ^ns^ *p* > 0.05.

### 3.11 HuR Plays Pivotal Role in HCC Resist to Lenvatinib on Xenograft Tumours in Mice In Vivo

This study clarified the core regulatory mechanism of the HuR‐GPX4 axis in lenvatinib resistance in liver cancer through a multi‐level in vivo and in vitro experimental system. Animal experiments showed that there were no significant differences in tumour volume, weight, and growth rate between the Bel7404‐R group and Bel7404‐R‐NC group, but the tumour growth of the parental cells in the Bel7404 group was significantly inhibited (*p* < 0.05). After interfering with HuR expression, the tumour volume in the Bel7404‐R‐shHuR group was significantly smaller than that in the Bel7404‐R group (*p* < 0.05), and the efficacy of lenvatinib was significantly enhanced (Figure 11A–C). The rate of change in tumour volume was the smallest, suggesting that the absence of HuR could reverse the drug‐resistant phenotype. Molecular mechanism studies indicated that Western blotting confirmed that the protein expression of HuR and GPX4 in the Bel7404‐R‐shHuR group was lower than that in the Bel7404‐R group (*p* < 0.05, *p* < 0.001) (Figure 11D); Further IHC showed that the expression of Ki67, HuR, EGFR, VEGFR, MDR1, and MRP1 in the Bel7404‐R group was significantly upregulated (*p* < 0.05), while shHuR in Bel7404 cells could simultaneously downregulate these drug‐resistant related markers. Notably, GPX4 was highly expressed in the drug‐resistant group and NC group, but significantly decreased in the parental cells and shHuR group (*p* < 0.001) (Figure 11E), which is consistent with the in vitro data. Comprehensive analysis indicated that HuR drives drug resistance through a dual mechanism: on the one hand, it up‐regulates GPX4 to inhibit ferroptosis (regulating Fe^2+^/ROS/MDA/GSH homeostasis); on the other hand, it activates the EGFR/VEGFR signalling pathway and enhances the drug excretion function mediated by MDR1/MRP1, jointly maintaining the proliferation and survival of tumour cells. Figure 11F shows a schematic diagram of how HuR‐GPX4 regulates intracellular protein functions and gene expression, leading to liver cancer cell tolerance to lenvatinib.

**FIGURE 11:**
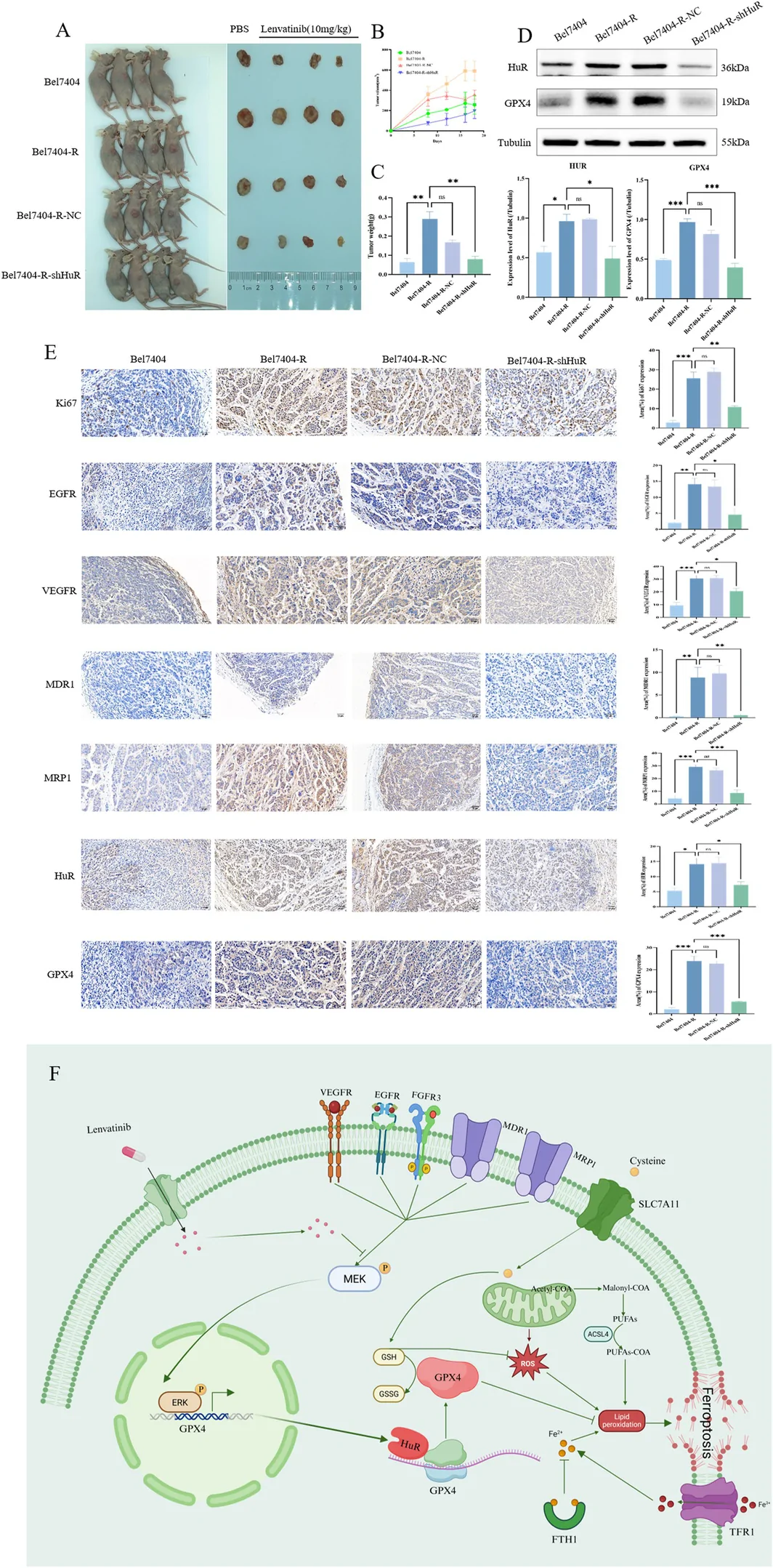
The effect of the HuR‐GPX4 axis in the growth of HCC and lenvatinib‐resistant related molecules in xenograft tumour in mice. (A) Tumour size in mice of the Bel7404, Bel7404‐R, Bel7404‐R‐NC, and Bel7404‐R‐shHuR xenograft groups. (B, C) Statistical analysis of tumour volume and weight. (D) The expression of HuR and GPX4 in the tumour tissues of BALB/c nude mice was detected by Western blotting; the bar chart on the right shows the statistics of the scanning grey values of the protein bands. (E) The expressions of Ki67, EGFR, VEGFR, MDR1, MRP1, HuR, and GPX4 were analysed by immunohistochemistry; the bar chart on the right shows the statistical analysis of the strength of immunohistochemical positivity. (F) A working model of how the HuR‐GPX4 axis inhibits ferroptosis and confers HCC resistance to the treatment of lenvatinib; the length of the scale is 50 μm. The proposed model in (F) is a working hypothesis based on the current experimental data; direct binding between HuR and GPX4 mRNA remains to be confirmed by future studies. Statistical analysis was performed as described in Methods. *p < 0.05; **p < 0.01; ***p < 0.001; ^ns^ *p* > 0.05.

## 4 Discussion

HCC is one of the most common primary malignant tumours worldwide. In the early stages, it can be treated by surgical resection. However, owing to the lack of obvious symptoms, most patients are diagnosed at an advanced stage and do not meet the conditions for surgical treatment [3, 4, 19]. In the advanced stage, treatment can be performed using the multi‐target tyrosinase inhibitor lenvatinib, thereby prolonging the median survival period of patients [6]. Lenvatinib, a first‐line drug for the treatment of advanced liver cancer, has better efficacy and tolerability than sorafenib, which is also a first‐line drug for liver cancer treatment [7]. However, the efficacy and duration of action of lenvatinib are limited by drug resistance, and the development of drug resistance is the main obstacle leading to the failure of lenvatinib treatment in HCC patients [20]. Therefore, there is an urgent need to explore the potential mechanism of lenvatinib resistance to provide a theoretical basis for prolonging the therapeutic effect of lenvatinib and improving the survival period of patients. This study explored the relationship between ferroptosis and lenvatinib resistance and revealed the key roles of HuR and GPX4 in liver cancer resistance. Inhibition of HuR increases the sensitivity of HCC cells to lenvatinib. Mechanistically, in the treatment with lenvatinib, HuR inhibits ferroptosis of HCC cells by regulating the expression of GPX4, further suppressing the efficacy of lenvatinib and thereby increasing the tolerance of HCC cells to lenvatinib. Furthermore, in a nude mouse xenograft model treated with lenvatinib, knockdown of HuR (using shRNA) significantly enhanced the sensitivity of HCC to lenvatinib treatment, indicating that HuR may be a novel intervention target for overcoming lenvatinib resistance in HCC.

As an RNA‐binding protein, HuR promotes the translation of various mRNAs into their corresponding proteins by regulating the stability of these mRNAs and exerting corresponding biological functions [21]. HuR is involved in proliferation, migration, and drug resistance of tumour cells [22, 23]. As a key regulatory factor of ferroptosis, GPX4 protects cells from the effects of ferroptosis by reducing the accumulation of lipid peroxides [24]. Therefore, high expression of HuR and GPX4 may jointly promote the malignant phenotype and drug resistance of HCC. To explore the relationship between HuR and GPX4 in liver cancer, we analysed the expression of HuR and GPX4 in the TCGA database. The results showed that HuR and GPX4 expressions were upregulated in liver cancer. The expression of HuR and GPX4 in all tumours, including HCC, was analysed using the timer database. The results showed that the expression of HuR and GPX4 in cancer tissues of various tumours was higher than that in adjacent normal tissues, and the same situation also occurs in liver cancer. To verify the above results, we collected tumour and adjacent tissues from 30 patients with HCC in clinical practice and fabricated immune tissue microarrays. The co‐expression of HuR and GPX4 in liver cancer was detected by multiplex fluorescence staining of the tissue microarrays. The results showed that the expression levels of HuR and GPX4 in liver cancer tissues were higher than those in adjacent normal liver tissues, which is consistent with our database analysis. Furthermore, we conducted a correlation analysis of HuR and GPX4 using the TCGA database. The results showed a correlation between HuR and GPX4 expression levels.

To explore the potential molecular mechanism of lenvatinib resistance in HCC, HCC cell lines (Bel7404 and Huh7) were used for lenvatinib drug gradient screening, and lenvatinib‐resistant cell lines (Bel7404‐R and Huh7‐R) were successfully constructed. The successful establishment of the drug‐resistant cell model was verified by comparing the half‐maximal inhibitory concentration (IC_50_) differences between drug‐resistant cells and parental cells, and by combining phenotypic analyses of cell apoptosis rate, proliferation ability, and migration ability. Lenvatinib can inhibit the mitogen‐activated protein kinase (MAPK) cascade by blocking the activities of VEGFR1‐VEGFR3, FGFR1‐FGFR4, PDGFRα, and RET, thereby suppressing tumour angiogenesis and cancer cell proliferation [8]. In this study, we detected the expression of VEGFR1 and FGFR3 in drug‐resistant HCC and parental cells. The results showed that the expression of VEGFR1 and FGFR3 was upregulated in drug‐resistant HCC cells, indicating that drug‐resistant HCC cells may reduce the inhibitory effect of lenvatinib and enhance tolerance by promoting the expression of lenvatinib target sites. More importantly, drug‐resistant cells exhibit characteristics of resistance to ferroptosis, including reduced levels of ferrous ions, lower content of reactive oxygen species, decreased lipid peroxidation, and increased levels of glutathione. Lipid peroxidation is caused by a complex process of lipid metabolism and is a marker of ferroptosis [25]. Studies have shown that polyunsaturated fatty acids (PUFA), formed through the lipid synthesis pathway, can significantly enhance the sensitivity of cells to ferroptosis. Acyl‐coA synthase long‐chain family member 4 (ACSL4) can damage the permeability and integrity of the cell membrane by catalysing the integration of PUFA into membrane phospholipids, which plays an important regulatory role in the occurrence of ferroptosis [26]. Transferrin receptor 1 (TFR1) mediates transferrin endocytosis. In ferroptosis‐susceptible cells, the expression of TFR1 is upregulated, enhancing the intracellular uptake of iron ions, and ultimately promoting the progression of ferroptosis [27]. Ferritin is a 24‐polymer protein complex composed of two subunits: a heavy chain (FTH1) and a light chain (FTL). Its structure is hollow and spherical, and it is capable of accommodating an inorganic iron core. The FTH1 subunit exhibits iron oxidase activity and can catalyse the oxidation of divalent iron (Fe^2+^) to trivalent iron (Fe^3+^). FTH1 stores excess Fe^2+^ in the cell. Preventing ferroptosis [28]. Member 7 of solute carrier family 11 (SLC7A11) is a component of the important intracellular antioxidant system Xc‐ and is the main mechanism by which cells defend themselves against ferroptosis [29]. In our study, the expression of ACSL4, TFR1, FTH1, and SLC7A11 in drug‐resistant HCC cells and parental cells was detected by immunofluorescence. We found that, compared with parental cells, the protein expression of ACSL4 and TFR1 in drug‐resistant HCC cells decreased, while the expression of FTH1 and SLC7A11 was upregulated. These results suggested that ferroptosis may be involved in the molecular resistance of hepatoma cells to lenvatinib. Recent studies have further expanded the landscape of ferroptosis regulation in hepatocellular carcinoma (HCC). Hiromatsu et al. demonstrated that transferrin receptor (TFRC) expression correlates with ferroptosis sensitivity and therapeutic vulnerability in HCC, highlighting the importance of iron metabolism in determining treatment response [30]. In parallel, Toshida et al. reported that TIGAR, a metabolic regulator involved in the pentose phosphate pathway, influences HCC prognosis through its dual effects on ferroptosis and the immune microenvironment [31]. These findings collectively underscore that ferroptosis modulation in HCC is a complex, multi‐faceted process involving iron uptake, lipid peroxidation, and metabolic reprogramming. Our study identifies the HuR‐GPX4 axis as a novel mechanism that confers lenvatinib resistance by inhibiting ferroptosis. Notably, the upregulation of GPX4 by HuR in drug‐resistant HCC cells leads to reduced ferrous iron, ROS, and lipid peroxidation—key features that are consistent with the ferroptosis‐resistant phenotype linked to TFRC dysregulation. Moreover, the connection between metabolic factors (e.g., TIGAR) and ferroptosis resistance further supports the notion that targeting the HuR‐GPX4 axis could serve as a complementary strategy to overcome lenvatinib resistance, potentially in combination with agents that modulate iron or redox homeostasis. Thus, our work integrates into and extends the growing body of evidence positioning ferroptosis as a central vulnerability in HCC therapy.

Combining the bioinformatics molecules and the results of multiplex tissue immunofluorescence staining in human liver cancer samples, we first detected the expression of HuR in liver cancer cell lines and normal liver cell line and found that HuR was significantly highly expressed in liver cancer cell lines than in normal liver cell line. HuR expression was detected in drug‐resistant and parental cells using Western blotting, qRT‐PCR, and cell immunofluorescence assays. The results showed that HuR expression in drug‐resistant HCC cells was significantly higher than that in parental cells. We constructed drug‐resistant HCC cell lines that interfered with HuR expression in vitro. Through CCK8, scratch assay, and plate cloning experiments, we observed that the sensitivity to lenvatinib increased while interfering with the expression of HuR in drug‐resistant HCC cell lines, and intracellular ferroptosis‐related indicators (such as ferrous ions, ROS, and lipid peroxidation levels) significantly increased, and the GSH level decreased. Further analysis was conducted to determine whether HuR promotes the resistance of HCC to lenvatinib by increasing its cytoplasmic content to regulate the expression levels of downstream protein mRNAs related to ferroptosis resistance. Nucleoplasmic separation showed that HuR expression was increased in the cytoplasm. Combined with the results of the bioinformatics analysis, it was suggested that HuR might affect the expression of GPX4 by regulating the stability or translation of mRNA in the cytoplasm. Western blotting and immunofluorescence results confirmed that after HuR knockdown, the expression of GPX4 was significantly reduced. GPX4 was overexpressed in shHuR cells and partially restored the IC_50_, proliferation, and migration abilities of drug‐resistant HCC cells, reduced intracellular ferroptosis‐related indicators (reactive oxygen species, ferrous ion levels, and lipid peroxidation levels), and increased GSH content. These results indicate that HuR reduces ferroptosis by stimulating the expression of GPX4, thereby promoting drug resistance in liver cancer. However, this study has several limitations. First, while our results suggest that HuR may influence ferroptosis by regulating GPX4 expression, we have not yet experimentally confirmed a direct interaction between HuR and GPX4 mRNA. Further studies employing techniques such as RNA immunoprecipitation (RIP), RNA pull‐down, or luciferase reporter assays are needed to verify this direct regulatory mechanism. Second, it should be acknowledged that this study did not employ classic ferroptosis inhibitors (such as ferrostatin‐1 or liproxstatin‐1) or iron chelators for rescue experiments. Although our data indicate that HuR regulates GPX4 expression and subsequently affects ferroptosis‐related phenotypes, the absence of these pharmacological interventions limits our ability to definitively establish ferroptosis as the primary downstream mechanism. Utilizing such inhibitors in future work would help to further consolidate the mechanistic link between HuR and ferroptosis in lenvatinib resistance. Additionally, it should be noted that the subcutaneous xenograft model used in this study can only partially recapitulate the pharmacological background, as it fails to fully mimic the tumour microenvironment and the metastatic niche of liver cancer. Future studies employing orthotopic tumour models are warranted to validate these findings in a setting that more closely resembles the clinical context, which would significantly strengthen the translational relevance of our conclusions. Fourth, in the GPX4 overexpression experiments, we observed a mild increase in HuR expression in some resistant cell lines (Figure 8A,B), suggesting a possible feedback loop from GPX4 to HuR. Whether GPX4 directly or indirectly regulates HuR expression requires further investigation, for example by knocking down GPX4 and examining HuR levels. Due to time and cell availability constraints, we did not perform these experiments in the current study, but we plan to address this question in future work.

## 5 Conclusions

This study found that HuR has the biological function of stimulating the expression of GPX4. The HUR‐GPX4 axis upregulates the expression and activity of VEGFR1‐3, a target of lenvatinib, and blocks lenvatinib to inhibit the tyrosine protein kinase signalling pathway. Moreover, HuR promotes the expression of GPX4, which causes liver cancer cells to resist ferroptosis‐induced cell death. This study found that the HuR‐GPX4 axis is an important target for lenvatinib tolerance in liver cancer cells. Blocking the HuR‐GPX4 axis and combining it with lenvatinib treatment is an effective strategy for solving the problem of liver cancer cell tolerance to lenvatinib treatment.

## Author Contributions

**Siren Feng:** conceptualization, investigation, writing – original draft, methodology, formal analysis, data curation. **Fang Wang:** conceptualization, investigation, writing – original draft, data curation. **Huayi Wu:** conceptualization, investigation, writing – original draft, methodology. **Xinran Lin:** conceptualization, investigation, data curation, resources. **Xue Qi:** conceptualization, investigation, formal analysis. **Kun Liu:** conceptualization, investigation, project administration, data curation. **Qiushi Yin:** conceptualization, investigation, resources, data curation. **Bo Lin:** conceptualization, investigation, data curation, project administration, funding acquisition. **Mengsen Li:** conceptualization, funding acquisition, writing – review and editing, supervision, project administration, investigation. **Mingyue Zhu:** conceptualization, funding acquisition, writing – review and editing, supervision, investigation.

## Funding

This study was supported by the National Natural Science Foundation of China (Nos. 82460602, 82560459, 82060514, and 82573045), the Joint Program on Health Science and Technology Innovation of Hainan Province (WSJK2025MS132, WSJK2024MS158), the Natural Science Foundation of Hainan Province (Nos. 822QN311 and 820QN403), the Hainan Provincial Science and Technology Special Fund (No. ZDYF2021SHF222), Nanhai Junior Talent Program of the Hainan Provincial Health Commission (No. NHXX‐WJW‐2023014), and Hainan Medical University Talent Launch Fund (No. XRC220017).

## Ethics Statement

The study was approved by the Scientific Research Ethics Committee of Hainan Medical University.

## Conflicts of Interest

The authors declare no conflicts of interest.

## Data Availability

The datasets generated and/or analysed during the current study are available in the TIMER database (http://timer.cistrome.org/) for analysis the expression of HuR and GPX4 in tumour and normal tissues. Other data availability is not applicable in this study.
